# Multi-adaptive event-triggered consensus of positive multi-agent systems using pinning strategy

**DOI:** 10.1371/journal.pone.0344567

**Published:** 2026-04-16

**Authors:** Qingbo Li, Lishuo Dong, Haoyue Yang

**Affiliations:** 1 School of Mathematics and Statistics, Hainan Normal University, Haikou, China; 2 School of Information and Communication Engineering, Hainan University, Haikou, China; Northeastern University, China, CHINA

## Abstract

This paper investigates the multi-adaptive event-triggered consensus of positive multi-agent systems. First, two classes of event-triggered mechanisms are designed for leaders and followers, respectively. A multi-adaptive event-triggered pinning control protocol is proposed by virtue of the presented mechanisms. Compared to existing event-triggered pinning strategies, the proposed method further reduces communication costs and resource consumption. By using the matrix decomposition technique and linear programming approach, the gain matrices of the control protocol and sufficient conditions are constructed to ensure the positivity and consensus of the systems. The multi-adaptive event-triggered pinning control protocol is then extended to observer-based control scenarios, where pinning observers for both the leader and follower are designed separately, further reducing the update frequency of the observer. Moreover, the adaptive technique and event-triggered mechanism are combined to reduce the length of the triggering interval, thereby further conserving the overall system resources. Meanwhile, a bound for the minimum event-triggering interval is derived, which analytically proves the exclusion of Zeno behavior. Finally, the effectiveness of the results is verified via an illustrative example and comparative simulations.

## 1 Introduction

Multi-agent systems (MASs) are composed of a series of interacting agents that communicate and collaborate with each other to accomplish large-scale and complex tasks [1]. They are widely applied in power engineering [2], zero-energy community [3], energy optimisation [4], and so on. The consensus issue is a core issue in MASs. The literature [5] discussed hybrid MASs with continuous and discrete dynamic agents and proposed three consensus protocols. The literature [6] and [7] investigated the consensus issue of MASs with fixed directed graph and undirected graph, respectively. However, in practical applications, the state of most systems may not be directly accessible. The observer can estimate these unmeasurable states by using the input and output information of the systems, which enables a comprehensive monitoring of the system. An observer-based dynamic event-triggered semi-global two-by-two consensus problem was addressed in [8]. The literature [9] constructed nonlinear observers to estimate the dynamics of unmeasurable subjects and used the Lyapounov-Krasovskii functional for analyzing the consensus.

In practical engineering, there exists a class of MASs with nonnegative states and they are called positive MASs (PMASs) [10,11]. For the consensus of PMASs, existing results [5–9] cannot be directly applied to PMASs due to the nonnegative property of the system. A convex programming algorithm was presented in [12] for designing a consensus protocol of PMASs. The literature [13] derived sufficiently necessary conditions for the consensus of all agents under the positivity constraint. In [14], the positive consensus was presented for homogeneous PMASs and a set of sufficient conditions were derived in the framework of linear programming (LP). The positive consensus was also addressed in [15] for MASs with multiple time-varying time delays and switching topologies. The literature [16] employed LP and copositive Lyapunov function (CLF) to handle the positive consensus problem of PMASs, which further reduces the computational burden of the obtained conditions. Then, the literature [17] and [18] designed nonlinear and proportional-integral observers respectively for the state unmeasurability problem of PMASs. It is shown that CLF is more suitable for PMASs than traditional quadratic Lyapunov function [10,12]. Meanwhile, LP can reduce the computational burden of the positivity and consensus conditions of PMASs. It is important to note that the above results regarding PMASs all ignore the fact that real-world resources are limited. This means that using a time-triggered approach for controller updates would waste a significant amount of resources.

In practice, the number of agents in MASs is large. If the leader-follower control strategy is used, a large amount of resources will be required to control each agent. This will lead to high consumption of resources and low efficiency. On the contrary, the pinning control strategy proposed in [19,20] is able to significantly reduce the required resources and improve efficiency by controlling some of agents. The literature [20–22] investigated the consensus of MASs based on pinning control. A pinning control approach was proposed in [21] to address the practical consensus problem of multi-agent systems with quantized communication, wherein an event-triggered mechanism based on local quantized state information was designed to reduce the communication burden. Based on the different information sources received by agents, a class of intermittent adaptive pinning control protocols was proposed in [22], which can reduce the communication cost effectively. The literature [23] addressed the event-triggered pinning consensus control problem for multi-agent networks under limited communication capabilities by employing an approach based on discontinuous distributed information exchange. Although pinning control is a well-established technique for general multi-agent systems, its extension to PMASs presents distinct and non-trivial challenges. Existing frameworks often fail to explicitly preserve positivity or exploit the specific structure of PMASs, leading to a notable shortage of applicable results. Therefore, the development of a dedicated pinning control framework, that systematically addresses state constraints and distributed coordination-emerges as a central, open problem in advancing the control of PMASs.

Meanwhile, how to utilize the resources properly is an issue that needs to be considered in practical application. Event-triggered mechanism (ETM) is a common method of solving this issue. The literature [23] used a static ETM to reduce the frequency of controller updates. Compared with the static ETM, the dynamic ETM is flexible and can be triggered by the change of system state. Dynamic ETM was used in [24–26]. An adaptive saturated threshold ETM was designed in [26]. The transient and steady-state performance of the nonlinear Markov jump system was guaranteed by the improved adaptive global preassigned performance control method. Recently, the topic of event-triggered control for positive systems has attracted the research interest and attention of many researchers [27–31]. The literature [27] proposes a fixed-threshold ETM for observer design of positive systems to save network bandwidth. Subsequently, [29] addressed the control problem of positive Markov jumping systems by designing a static ETM using the 1-norm. Furthermore, an adaptive event-triggered control approach for positive semi-Markov jumping systems was proposed in [31]. However, it should be noted that the aforementioned event-triggered mechanisms for positive systems still exhibit certain limitations, such as the trigger threshold being fixed and only considering the ETM of a single channel. These limitations prevent the maximization of data update frequency reduction, thereby hindering resource conservation. To further save resources, double ETM was investigated in [32–34]. Double event-triggered mechanisms have different triggering conditions and can work independently. The literature [33] combined dynamic and static event-triggered mechanisms. However, the ETM of general systems cannot be directly applied for PMASs. The literature [35] designed a fuzzy event-triggered control protocol of T-S fuzzy PMASs by using event-triggered compensation information. The double ETM was extended to PMASs in [18]. The triggered strategy in [32–34] was investigated for control update and communication between agents but it did not refer to observer and controller. Moreover, the literature [11,18,23] only set one leader. When the practical application contains more than one leader, how to reduce the consumption of leaders also needs to be considered. Based on the above points, some questions arise: How to construct pinning control framework for PMASs? How to build event-triggered control protocol and event-triggered observer for PMASs? How to present the ETM for the leaders? These questions remain to be discussed.

Inspired by the above analysis and discussion, this paper focuses on the pinning control and the design of multi-adaptive ETM for PMASs. The main contributions of this paper can be summarized in the following three aspects:

(i) Double adaptive ETM are proposed for multi-leaders and followers, respectively. Compared to existing event-triggered schemes that only focus on followers, the proposed method significantly reduces the communication cost and resource consumption of the entire system.
(ii) A pinning consensus control protocol is designed to achieve practical consensus in PMASs with multiple leaders. This ensures that the failure of any agent does not affect the entire system and enhances system stability.
(iii) Unlike quadratic Lyapunov functions and linear matrix inequalities, the computationally simpler and lower-complexity CLF and LP methods are employed to analyze the consensus and the design of the corresponding gain matrices.

The paper is organized as follows: Section 2 represents the problem statement, main results are presented in Section 3, Section 4 discusses quantization effects, Section 5 gives an example, and Section 6 summarizes the conclusions and provides the future work.

**Notation R**^*n*^ and ${𝐑}^{n\times r}$ denote *n*-dimensional vectors and n×r matrices, respectively. ${\mathbb{N}}^{+}$ is the set of positive integers. $A^{⊤}$ is the transpose of *A*. *I*_*n*_ is an *n*-dimensional identity matrix. $1_{n\times n}$ and $1_{n}$ are matrices and vectors with all elements being 1. $1_{n}^{\left(i\right)}=\{\underset{i-1}{\underbrace{0,\ldots ,0}},1,\underset{n-i}{\underbrace{0,\ldots ,0}}\}$. All elements greater than (greater than or equal to) 0 are expressed as ≻0 (⪰0). All elements less than (less than or equal to) 0 are ≻0 (⪰0). The 1-norm of an *n*-dimensional vector *x* is denoted as $\|x{\|}_{1}=\sum_{i=1}^{n}|x_{i}|$, where *x*_*i*_ is the *i*th element of *x*. The symbol ⊗ is the Kronecker product.

## 2 Problem formulation and preliminaries

In this section, some preliminaries are introduced for graph theory and MASs.

Consider a graph 𝒢=(𝒱,ℰ) with *n* nodes. Let $𝒱=\{v_{1},v_{2},\ldots ,v_{N}\}$ denotes the vertex set, and ℰ={(i,j)|i,j=1,2,…,N} represents the edge set, where (i,j)∈ℰ if and only if nodes *i* and *j* have information interaction. It also means that agent *j* is an inner neighbor of agent *i*. If (i,j)∈ℰ⇔(j,i)∈ℰ, the communication topology is an undirected graph, otherwise it is a directed graph. If each agent can be regarded as a root agent, the graph is strongly connected. If this graph is a directed graph, it is called a strongly connected graph. In this paper, a strongly connected graph is used. The interactions between vertexes *i* and *j* are described by the adjacency matrix $𝒜=\{a_{ij}\}\in {𝐑}^{N\times N}$, where *a*_*ij*_ = 1 if (i,j)∈ℰ and *a*_*ij*_ = 0 otherwise. $D=diag\{d_{1},d_{2},\ldots ,d_{N}\}$ is the degree matrix of the graph 𝒢, where $d_{i}=\sum_{j=1}^{n}a_{ij}$. The Laplacian matrix is defined as ℒ=𝒟−𝒜.

### 2.1 Problem formulation

Consider the following MASs:


$$\begin{array}{c} {\dot{x}}_{i}(t)=Ax_{i}(t)+Bu_{i}(t),i\in ℳ,\\ y_{i}(t)=Cx_{i}(t), \end{array}$$ (1)

where $x_{i}(t)=(x_{i1},x_{i2},\ldots ,x_{in}{)}^{⊤}\in {𝐑}^{n}$, $u_{i}(t)\in {𝐑}^{r}$, $y_{i}(t)\in {𝐑}^{q}$ represent the state, the control input, and the output, respectively; $A\in {𝐑}^{n\times n}$, $B\in {𝐑}^{n\times r}$, $C\in {𝐑}^{q\times n}$. It is assumed that *A* is Metzler, B⪰0 and C⪰0. Consider the dynamics of multiple leaders as


$$\begin{array}{c} {\dot{x}}_{0k}(t)=A_{0}x_{0k}(t)+B_{0}u_{0k}(t),k=1,\cdots ,M,\\ y_{0k}(t)=C_{0}x_{0k}(t), \end{array}$$ (2)

where $x_{0k}(t)=(x_{0k1},x_{0k2},\ldots ,x_{0kn}{)}^{⊤}\in {𝐑}^{n}$, *u*_0*k*_(*t*), and $y_{i}(t)\in {𝐑}^{q}$ are the leader state, *t*he leader control input, the leader output, respectively; *A*_0_ is Metzler matrices, $B_{0}⪰0$, and $C_{0}⪰0$.

Next, some definitions and lemmas are introduced.

**Definition 1** [36] A system is positive if its state and outputs are nonnegative for any nonnegative input and output.

**Definition 1** The practical consensus of the system (1) is achieved if the following conditions are satisfied:

(i) $\lim_{t\to \infty }\|x_{0k}(t)-x_{0}^{*}\|<ð$ holds for k=1,⋯,M;
(ii) $\lim_{t\to \infty }\|x_{i}(t)-x_{0}^{*}\|<∁$ holds for i=1,⋯,N, where $x_{0}^{*}⪰0$, ð>0, and ∁>0.

**Lemma 1** [36] System (1) is positive if *A* is a Metzler matrix and B⪰0, C⪰0.

**Lemma 2** [36] For a positive system $\dot{x}(t)=Ax(t)$, the following conditions are equivalent:

(i) The system matrix *A* is Hurwitz;
(ii) The system is stable;
(iii) There exists a vector $v\in {𝐑}^{n}$ with v≻0 such that $A^{⊤}v≺0$.

**Lemma 3** [36] A matrix *A* is a Metzler matrix if there exists a constant ϵ such that A+ϵI⪰0.

## 3 Main result

In this section, the consensus of systems (1) and (2) is introduced. First, event-triggered control protocols are established for leaders and followers to address the consensus of PMASs. Then, event-triggered observers are designed for leaders and followers, respectively. Based on the designed observers, event-triggered control protocols are proposed to solve the consensus of PMASs.

### 3.1 Pinning control

To achieve the consensus, a pinning control approach is used in this paper. Only some agents need to have information interaction with the target $x_{0}^{*}$. Therefore, the pinning controller can be designed as:


$$\begin{array}{c} u_{0k}(t)=K_{01}z_{0k}(t_{p}^{k})+K_{02}(x_{0k}(t)-x_{0}^{*})+K_{03}x_{0k}(t)+Gx_{0}^{*},\;k=1,2,\ldots ,Q,\\ u_{0k}(t)=K_{01}z_{0k}(t_{p}^{k})+K_{03}x_{0k}(t)+Gx_{0}^{*},\;k=Q+1,Q+2,\ldots ,M, \end{array}$$ (3)

where $t_{p}^{k}$ is the sequence of communication triggers for leader *k*, *K*_01_, *K*_02_, *K*_03_, and *G* are gain matrices. The pinning controller of the followers’ system is described as:


$$\begin{array}{c} u_{i}(t)=K_{1}z_{i}(t_{\sigma }^{i})+K_{2}\sum_{k=1}^{M}c_{ik}(x_{i}(t)-x_{0k}(t))+K_{3}x_{i}(t)+Hx_{0}^{*},\;i=1,2,\ldots ,M,\;\\ u_{i}(t)=K_{1}z_{i}(t_{\sigma }^{i})+K_{3}x_{i}(t)+Hx_{0}^{*},\;i=M+1,M+2,\ldots ,N, \end{array}$$ (4)

where $t_{\sigma }^{i}$ is the sequence of communication triggers for follower *i* and $K_{1},K_{2},K_{3}$, and *H* are gain matrices. If leader and follower have information interaction, *c*_*ik*_ = 1; otherwise *c*_*ik*_ = 0. The following state thresholds *z*_0*k*_(*t*) and *z*_*i*_(*t*) are defined as:


$$\begin{array}{c} z_{0k}(t)=\sum_{s\in {ℳ}_{k}}b_{ks}(x_{0k}(t)-x_{0s}(t)),\\ z_{i}(t)=\sum_{j\in {𝒩}_{i}}a_{ij}(x_{i}(t)-x_{j}(t)), \end{array}$$ (5)

where ${ℳ}_{k}$ is the in-neighbor set of leader *k*, and *b*_*rs*_ is the *r*th row and *s*th column component of the leader adjacency matrix in (1), and *z*_*i*_(*t*) has the same properties as *z*_0*k*_(*t*). Define *t*he tracking errors *e*_0*k*_(*t*) and *e*_*i*_(*t*) as:


$$\begin{array}{c} e_{0k}(t)=x_{0k}(t)-x_{0}^{*}, \end{array}$$ (6)

and


$$\begin{array}{c} e_{i}(t)=x_{i}(t)-x_{0}^{*}, \end{array}$$ (7)

respectively. Define the sampling errors $\phi_{k}(t)$ and $\varphi_{i}(t)$ by


$$\begin{array}{c} \phi_{k}(t)=z_{0k}(t_{p}^{k})-z_{0k}(t),\;\varphi_{i}(t)=z_{i}(t_{\sigma }^{i})-z_{i}(t). \end{array}$$ (8)

The event-triggered conditions are constructed as:


$$\begin{array}{c} \|\phi_{k}(t){\|}_{1}>\alpha_{k}(t)\|e_{0k}(t){\|}_{1},\;\|\varphi_{i}(t){\|}_{1}>\nu_{i}(t)\|e_{i}(t){\|}_{1}, \end{array}$$ (9)

where


$$\begin{array}{c} {\dot{\alpha }}_{k}(t)=(\Lambda_{k}-\alpha_{k}(t))(\|\phi_{k}(t){\|}_{1}-\overset{―}{\alpha }\|e_{0k}(t){\|}_{1}),\\ {\dot{\nu }}_{i}(t)=(\Gamma_{i}-\nu_{i}(t))(\|\varphi_{i}(t){\|}_{1}-\overset{―}{\nu }\|e_{i}(t){\|}_{1}), \end{array}$$ (10)

with $0<\alpha_{k}(t_{0})<\Lambda_{k}<\overset{―}{\alpha }$, $0<\nu_{i}(t_{0})<\Gamma_{i}<\overset{―}{\nu }$, $\alpha_{k}(t_{0})$ and $\nu_{i}(t_{0})$ are initial conditions, and $\Lambda_{k},\Gamma_{i},\overset{―}{\alpha },\overset{―}{\nu }$ are known constants. In the initial condition, it satisfies that $\Lambda_{k}-\alpha_{k}(t_{0})>0$ and $\Gamma_{i}-\nu_{i}(t_{0})>0$. If $\|\phi_{k}(t){\|}_{1}-\overset{―}{\alpha }\|e_{0k}(t){\|}_{1}<0$ and $\|\varphi_{i}(t){\|}_{1}-\overset{―}{\nu }\|e_{i}(t){\|}_{1}<0$, the event-triggered conditions are not satisfied. Then, if follows that ${\dot{\alpha }}_{k}(t)<0$ and ${\dot{\nu }}_{i}(t)<0$. Furthermore, it is obtained that $0<\alpha_{k}(t)<\overset{―}{\alpha }$ and $0<\nu_{i}(t)<\overset{―}{\nu }$. When ${\dot{\alpha }}_{k}(t)=0$ and ${\dot{\nu }}_{i}(t)=0$, the dynamic event-triggered mechanism becomes the static one.

**Remark 1** In references [28–31,37], the event-triggered control problem for various systems has been addressed. At the same time, a variety of event-triggered mechanisms have been proposed, including static event-triggered mechanism [28,29], single adaptive event-triggered mechanism [31], and switching threshold event-triggered mechanism [35]. This paper proposes a multi-adaptive event-triggering mechanism in (9) and (10) to simultaneously reduce the state update frequency of both the leader and follower agents. It not only improves the fixed threshold in [28,29] to an adaptive threshold, but also further reduces the state update frequency of the system compared to [31], thereby reducing resource consumption. The event-triggered mechanism based on the change of control input amplitude is proposed in [37]. Its advantage is that it avoids excessive triggering error caused by excessive control input amplitude by using threshold switching. And (9) and (10) are more suitable for the event-triggered control problem of positive systems compared to [37] and save more resources.

Next, the pinning control and adaptive event-triggered consensus are achieved for leaders and followers.

**Theorem 1** If there exist constants $\epsilon_{1}>0$, $\epsilon_{2}>0$, $\omega_{1}>0$, $\omega_{2}>0$, ${\mathbb{R}}^{n}$ vectors $v_{0}≻0$, $v_{1}≻0$, $v_{a}≻0$, $\beta_{ı}≺0$, $\gamma_{ı}≺0$, $\eta_{ı}≺0$, $\sigma_{ı}≻0$, $\delta_{ı}≺0$, $\zeta_{ı}≺0$, $\theta_{ı}≺0$, $\varsigma_{ı}≻0$, β≺0, γ≺0, η≺0, σ≻0, δ≺0, ζ≺0, θ≺0, ς≻0 such that


$$\begin{array}{c} 1_{r}^{⊤}B_{0}^{⊤}v_{0}A_{0}+\sum_{s\in {ℳ}_{k}}a_{ks}B_{0}\sum_{ı=1}^{r}1_{r}^{\left(ı\right)}\beta_{ı}^{⊤}+B_{0}\sum_{ı=1}^{r}1_{r}^{\left(ı\right)}\gamma_{ı}^{⊤}\\ +B_{0}\sum_{ı=1}^{r}1_{r}^{\left(ı\right)}\eta_{ı}^{⊤}+\overset{―}{\alpha }B_{0}\sum_{ı=1}^{r}1_{r}^{\left(ı\right)}\beta_{ı}^{⊤}1_{n\times n}+\epsilon_{1}I⪰0, \end{array}$$ (11a)

$$\begin{array}{c} 1_{r}^{⊤}B_{0}^{⊤}v_{0}A_{0}+\sum_{s\in {ℳ}_{k}}a_{ks}B_{0}\sum_{ı=1}^{r}1_{r}^{\left(ı\right)}\beta_{ı}^{⊤}+B_{0}\sum_{ı=1}^{r}1_{r}^{\left(ı\right)}\eta_{ı}^{⊤}\\ +\overset{―}{\alpha }B_{0}\sum_{ı=1}^{r}1_{r}^{\left(ı\right)}\beta_{ı}^{⊤}1_{n\times n}+\epsilon_{1}I⪰0, \end{array}$$ (11b)

$$\begin{array}{c} 1_{r}^{⊤}B^{⊤}vA+\sum_{j\in {𝒩}_{i}}a_{ij}B\sum_{ı=1}^{r}1_{r}^{\left(ı\right)}\delta_{ı}^{⊤}+B\sum_{ı=1}^{r}1_{r}^{\left(ı\right)}\zeta_{ı}^{⊤}\\ +B\sum_{ı=1}^{r}1_{r}^{\left(ı\right)}\theta_{ı}^{⊤}+\overset{―}{\nu }B\sum_{ı=1}^{r}1_{r}^{\left(ı\right)}\delta_{ı}^{⊤}1_{n\times n}+\epsilon_{2}I⪰0, \end{array}$$ (11c)

$$\begin{array}{c} 1_{r}^{⊤}B^{⊤}vA+\sum_{j\in {𝒩}_{i}}a_{ij}B\sum_{ı=1}^{r}1_{r}^{\left(ı\right)}\delta_{ı}^{⊤}+B\sum_{ı=1}^{r}1_{r}^{\left(ı\right)}\zeta_{ı}^{⊤}\\ +\overset{―}{\nu }B\sum_{ı=1}^{r}1_{r}^{\left(ı\right)}\delta_{ı}^{⊤}1_{n\times n}+\epsilon_{2}I⪰0, \end{array}$$ (11d)

$$\begin{array}{c} (1_{r}^{⊤}B_{0}^{⊤}v_{0}A_{0}+B_{0}\sum_{ı=1}^{r}1_{r}^{\left(ı\right)}\eta_{ı}^{⊤}+B_{0}\sum_{ı=1}^{r}1_{r}^{\left(ı\right)}\sigma_{ı}^{⊤})x_{0}^{*}⪰0, \end{array}$$ (11e)

$$\begin{array}{c} (1_{r}^{⊤}B^{⊤}vA+B\sum_{ı=1}^{r}1_{r}^{\left(ı\right)}\theta_{ı}^{⊤}+B\sum_{ı=1}^{r}1_{r}^{\left(ı\right)}\varsigma_{ı}^{⊤})x_{0}^{*}⪰0, \end{array}$$ (11f)

$$\begin{array}{c} A_{0}v_{0}+\sum_{s\in {ℳ}_{k}}a_{ks}\beta -\sum_{s\in {ℳ}_{k}}a_{sk}\beta +\gamma +\eta -\overset{―}{\alpha }1_{n\times n}\beta \\ -\zeta +\omega_{1}v_{0}≺0,\;k=1,2,\cdots ,M, \end{array}$$ (11g)

$$\begin{array}{c} A_{0}v_{0}+\sum_{s\in {ℳ}_{k}}a_{ks}\beta -\sum_{s\in {ℳ}_{k}}a_{sk}\beta +\eta -\overset{―}{\alpha }1_{n\times n}\beta \\ +\omega_{1}v_{0}≺0,\;k=M+1,M+2,\cdots ,N, \end{array}$$ (11h)

$$\begin{array}{c} Av+\sum_{j\in {𝒩}_{i}}a_{ij}\delta -\sum_{j\in {𝒩}_{i}}a_{ji}\delta +\zeta +\theta \\ -\overset{―}{\nu }1_{n\times n}\delta +\omega_{1}v≺0,\;i=1,2,\cdots ,M, \end{array}$$ (11i)

$$\begin{array}{c} Av+\sum_{j\in {𝒩}_{i}}a_{ij}\delta -\sum_{j\in {𝒩}_{i}}a_{ji}\delta +\theta -\overset{―}{\nu }1_{n\times n}\delta \\ +\omega_{1}v≺0,\;i=M+1,M+2,\cdots ,N, \end{array}$$ (11j)

$$\begin{array}{c} 1_{\emptyset }⊗A_{0}^{⊤}v_{0}+1_{\emptyset }⊗\eta +1_{\emptyset }⊗\sigma +1_{N}⊗A^{⊤}v+1_{N}⊗\theta \\ +1_{N}⊗\varsigma -\omega_{2}(1_{N}⊗v_{a})≺0, \end{array}$$ (11k)

$$\begin{array}{c} \beta_{ı}⪯\beta ,\gamma_{ı}⪯\gamma ,\delta_{ı}⪯\delta ,\zeta_{ı}⪯\zeta ,\eta_{ı}⪯\eta ,\theta_{ı}⪯\theta ,\sigma_{ı}⪯\sigma ,\varsigma_{ı}⪯\varsigma , \end{array}$$ (11l)

hold for ı=1,2,⋯,r, then under the leader’s control protocol (3) and the followers’ control protocol (4) with


$$\begin{array}{c} K_{01}=\frac{\sum_{ı=1}^{r}1_{r}^{\left(ı\right)}\beta_{ı}^{⊤}}{1_{r}^{⊤}B_{0}^{⊤}v_{0}},K_{02}=\frac{\sum_{ı=1}^{r}1_{r}^{\left(ı\right)}\gamma_{ı}^{⊤}}{1_{r}^{⊤}B_{0}^{⊤}v_{0}},K_{03}=\frac{\sum_{ı=1}^{r}1_{r}^{\left(ı\right)}\eta_{ı}^{⊤}}{1_{r}^{⊤}B_{0}^{⊤}v_{0}},G=\frac{\sum_{ı=1}^{r}1_{r}^{\left(ı\right)}\sigma_{ı}^{⊤}}{1_{r}^{⊤}B_{0}^{⊤}v_{0}},\\ K_{1}=\frac{\sum_{ı=1}^{r}1_{r}^{\left(ı\right)}\delta_{ı}^{⊤}}{1_{r}^{⊤}B^{⊤}v},K_{2}=\frac{\sum_{ı=1}^{r}1_{r}^{\left(ı\right)}\zeta_{ı}^{⊤}}{1_{r}^{⊤}B^{⊤}v},K_{3}=\frac{\sum_{ı=1}^{r}1_{r}^{\left(ı\right)}\theta_{ı}^{⊤}}{1_{r}^{⊤}B^{⊤}v},H=\frac{\sum_{ı=1}^{r}1_{r}^{\left(ı\right)}\varsigma_{ı}^{⊤}}{1_{r}^{⊤}B_{0}^{⊤}v_{0}}, \end{array}$$ (12)

the system (1) is positive and the systems (1) and (2) achieve the practical consensus, where $X_{0}^{*}=(\underset{M}{\underbrace{x_{0}^{*⊤},\ldots ,x_{0}^{*⊤}}}{)}^{⊤}$, $X^{*}=(\underset{N}{\underbrace{x_{0}^{*⊤},\ldots ,x_{0}^{*⊤}}}{)}^{⊤}$, and $\Phi =(1_{M},\underset{N-M}{\underbrace{0,\ldots ,0}})$.

**Proof:** From (9), it is obtained that


$$\begin{array}{c} \|\phi_{k}(t){\|}_{1}\leq \alpha_{k}(t)\|x_{0k}(t){\|}_{1},\;\|\varphi_{i}(t){\|}_{1}\leq \nu_{i}(t)\|e_{i}(t){\|}_{1}. \end{array}$$ (13)

For $x_{0k}(t_{0})⪰0$, $x_{i}(t_{0})⪰0$, it yields that $y_{0k}(t_{0})⪰0$ and $y_{i}(t_{0})⪰0$. Then,


$$\begin{array}{c} -\overset{―}{\alpha }1_{n\times n}e_{0k}(t_{0})⪯\phi_{k}(t_{0})⪯\overset{―}{\alpha }1_{n\times n}e_{0k}(t_{0}),\\ -\overset{―}{\nu }1_{n\times n}e_{i}(t_{0})⪯\varphi_{i}(t_{0})⪯\overset{―}{\nu }1_{n\times n}e_{i}(t_{0}), \end{array}$$ (14)

where $\sigma_{i}(t_{0})$ and $\zeta_{i}(t_{0})$ are initial conditions. By (2), (3), (5), (6), and (8), the following relation is considered. For k=1,2,⋯,Q, it is obtained that


$$\begin{array}{c} \begin{array}{cc} {\dot{e}}_{0k}(t) & \;=A_{0}e_{0k}(t)+B_{0}K_{01}(\phi_{k}(t)+\sum_{s\in {ℳ}_{k}}b_{ks}(e_{0k}(t)-e_{0s}(t)))\\ & \;\;+B_{0}K_{02}e_{0k}(t)+B_{0}K_{03}e_{0k}(t)+(A_{0}+B_{0}K_{03}+B_{0}G)x_{0}^{*}. \end{array} \end{array}$$ (15)

For k=Q+1,Q+2,⋯,M, it is obtained that


$$\begin{array}{c} \begin{array}{cc} {\dot{e}}_{0k}(t) & \;=A_{0}e_{0k}(t)+B_{0}K_{01}(\phi_{k}(t)+\sum_{s\in {ℳ}_{k}}b_{ks}(e_{0k}(t)-e_{0s}(t)))\\ & \;\;+B_{0}K_{03}e_{0k}(t)+(A_{0}+B_{0}K_{03}+B_{0}G)x_{0}^{*}. \end{array} \end{array}$$ (16)

Combining (1), (4), (5), (7), (8) and considering the following relation for i=1,2,⋯,M give


$$\begin{array}{c} \begin{array}{cc} {\dot{e}}_{i}(t) & \;=Ae_{i}(t)+BK_{1}(\varphi_{i}(t)+z_{i}(t))+BK_{2}\sum_{k=1}^{M}c_{ik}(e_{i}(t)-e_{0k}(t))\\ & \;\;+BK_{3}e_{i}(t)+(A+BK_{3}+BH)x_{0}^{*}. \end{array} \end{array}$$ (17)

For i=M+1,M+2,⋯,N, it yields that


$$\begin{array}{c} \begin{array}{cc} {\dot{e}}_{i}(t) & \;=Ae_{i}(t)+BK_{1}(\varphi_{i}(t)+z_{i}(t))+BK_{3}e_{i}(t)+(A+BK_{3}+BH)x_{0}^{*}. \end{array} \end{array}$$ (18)

Let $E_{0}(t)=(e_{01}^{⊤}(t),\ldots ,e_{0M}^{⊤}(t){)}^{⊤}$, $E(t)=(e_{1}^{⊤}(t),\ldots ,e_{N}^{⊤}(t))$, $\phi (t)=(\phi_{1}^{⊤}(t),\ldots ,\phi_{M}^{⊤}(t){)}^{⊤}$, and $\varphi (t)=(\varphi_{1}^{⊤}(t),\ldots ,\varphi_{N}^{⊤}(t))$. Then,


$$\begin{array}{c} \begin{array}{cc} {\dot{E}}_{0}(t) & \;=A_{M0}E_{0}(t)+(I_{M}⊗B_{0}K_{01})\phi (t)+(I_{M}⊗A_{0}\\ & \;\;+\;I_{M}⊗B_{0}K_{03}+I_{M}⊗B_{0}G)X_{0}^{*},\\ \dot{E}(t) & \;=-B_{K_{2}}E_{0}(t)+A_{N}E(t)+(I_{N}⊗BK_{1})\varphi (t)\\ & \;\;+\;(I_{N}⊗A+I_{N}⊗BK_{3}+I_{N}⊗BH)X^{*}, \end{array} \end{array}$$ (19)

where


$$\begin{array}{cc} A_{M0} & =I_{M}⊗A_{0}+{ℒ}_{0}⊗B_{0}K_{01}+\overset{―}{B_{0}K_{02}}+I_{M}⊗B_{0}K_{03},\\ A_{N} & =I_{N}⊗A+ℒ⊗BK_{1}+\overset{―}{BK_{2}}+I_{N}⊗BK_{3},\\ \overset{―}{B_{0}K_{02}} & =diag\{\underset{Q}{\underbrace{B_{0}K_{02},B_{0}K_{02},\ldots ,B_{0}K_{02}}},\underset{M-Q}{\underbrace{0,0,\ldots ,0}}\},\\ \overset{―}{BK_{2}} & =diag\{\underset{M}{\underbrace{BK_{2},BK_{2},\ldots ,BK_{2}}},\underset{N-M}{\underbrace{0,0,\ldots ,0}}\},\\ B_{K_{2}} & =(\begin{array}{cccc} c_{11}BK_{2} & c_{12}BK_{2} & \cdots & c_{1M}BK_{2}\\ c_{21}BK_{2} & c_{22}BK_{2} & \cdots & c_{2M}BK_{2}\\ \vdots & \cdots & \ddots & \vdots \\ c_{M1}BK_{2} & c_{M2}BK_{2} & \cdots & c_{MM}BK_{2} \end{array}). \end{array}$$

Let $\tilde{E}(t)=(E_{0}^{⊤}(t),E^{⊤}(t){)}^{⊤}$. It is obtained that


$$\begin{array}{c} \begin{array}{cc} & \;\dot{\tilde{E}}(t)=(\begin{array}{cc} \begin{array}{c} A_{M0} \end{array} & 0\\ -B_{K_{2}} & A_{N} \end{array})\tilde{E}(t)+(\begin{array}{c} I_{M}⊗B_{0}K_{01}\\ 0 \end{array})\phi (t)\\ & \;+(\begin{array}{c} 0\\ I_{N}⊗BK_{1} \end{array})\varphi (t)+(\begin{array}{cc} I_{M}⊗{\bar{A}}_{0} & 0\\ 0 & I_{N}⊗\bar{A} \end{array})(\begin{array}{c} X_{0}^{*}\\ X^{*} \end{array}), \end{array} \end{array}$$ (20)

where ${\bar{A}}_{0}=A_{0}+B_{0}K_{03}+B_{0}G$ and $\bar{A}=A+BK_{3}+BH$. With initial conditions $E_{0}(t_{0})⪰0,E(t_{0})⪰0$, it follows from (14) and (19) that


$$\begin{array}{c} \begin{array}{cc} {\dot{E}}_{0}(t_{0}) & \;⪰{\bar{A}}_{M0}E_{0}(t_{0})+(I_{M}⊗A_{0}+I_{M}⊗B_{0}K_{03})X_{0}^{*},\\ \dot{E}(t_{0}) & \;⪰-B_{K_{2}}E_{0}(t_{0})+{\bar{A}}_{N}E(t_{0})+(I_{N}⊗A+I_{N}⊗BK_{3})X^{*}, \end{array} \end{array}$$ (21)

where ${\bar{A}}_{M0}=I_{M}⊗A_{0}+{ℒ}_{0}⊗B_{0}K_{01}+\overset{―}{B_{0}K_{02}}+I_{M}⊗B_{0}K_{03}+\overset{―}{\alpha }I_{M}⊗B_{0}K_{01}1_{n\times n}$ and ${\bar{A}}_{N}=I_{N}⊗A+ℒ⊗BK_{1}+\overset{―}{BK_{2}}+I_{N}⊗BK_{3}+\overset{―}{\nu }I_{N}⊗BK_{1}1_{n\times n}$. According to (20) and (21), one has


$$\begin{array}{cc} \dot{\tilde{E}}(t_{0}) & ⪰(\begin{array}{cc} {\bar{A}}_{M0} & 0\\ -B_{K_{2}} & {\bar{A}}_{N} \end{array})\tilde{E}(t_{0})+(\begin{array}{cc} I_{M}⊗{\bar{A}}_{0} & 0\\ 0 & \begin{array}{c} I_{N}⊗\bar{A} \end{array} \end{array})(\begin{array}{c} X_{0}^{*}\\ X^{*} \end{array}), \end{array}$$ (22)

On the basis of (11a), (11b), and (12), we can get $A_{0}+\sum_{s\in {ℳ}_{k}}a_{ks}B_{0}K_{01}+B_{0}K_{02}+B_{0}K_{03}+\overset{―}{\alpha }B_{0}K_{01}1_{n\times n}$ and $A_{0}+\sum_{s\in {ℳ}_{k}}a_{ks}B_{0}K_{01}+B_{0}K_{03}+\overset{―}{\alpha }B_{0}K_{01}1_{n\times n}$ are Metzler by Lemma 3. Thus, ${\bar{A}}_{M0}$ is Metzler. Similarly, it follows from (11c), (11d), (12), and Lemma 3 that $A+\sum_{j\in {𝒩}_{i}}a_{ij}BK_{1}+\overset{―}{\nu }BK_{1}1_{n\times n}+BK_{2}+BK_{3}$ and $A+\sum_{j\in {𝒩}_{i}}a_{ij}BK_{1}+\overset{―}{\nu }BK_{1}1_{n\times n}+BK_{3}$ are Metzler. As a result, ${\bar{A}}_{N}$ is Metzler.

Using (11e), (11f) and (12) gives ${\bar{A}}_{0}x_{0}^{*}⪰0$ and $\bar{A}x_{0}^{*}⪰0$. Based on this, it is obtained that $(I_{M}⊗A_{0}+I_{M}⊗B_{0}K_{03})X_{0}^{*}⪰0$ and $(I_{N}⊗A+I_{N}⊗BK_{3})X^{*}⪰0$. Since ζ≺0, $BK_{2}≺0$, i.e., $-BK_{2}≻0$. By means of Lemma 1, we have $\dot{\tilde{E}}(t)⪰0$ for $\dot{\tilde{E}}(t_{0})⪰0$, it means that $\tilde{E}(t)⪰0$. Furthermore, for any initial state $\tilde{E}(t_{0})⪰0$, $\tilde{E}(t)⪰0$ is derived. Therefore, the system (20) is positive.

Next, the consensus of system (20) is considered. From (14) and (19), the following inequalities are deduced:


$$\begin{array}{c} \begin{array}{cc} {\dot{E}}_{0}(t) & \;⪯{\tilde{A}}_{M0}E_{0}(t)+(I_{M}⊗A_{0}+I_{M}⊗B_{0}K_{03}+I_{M}⊗B_{0}G)X_{0}^{*},\\ \dot{E}(t) & \;⪯-B_{K_{2}}E_{0}(t)+{\tilde{A}}_{N}E(t)+(I_{N}⊗A+I_{N}⊗BK_{3}+I_{N}⊗BH)X^{*}, \end{array} \end{array}$$ (23)

where ${\tilde{A}}_{M0}=I_{M}⊗A_{0}+{ℒ}_{0}⊗B_{0}K_{01}+\overset{―}{B_{0}K_{02}}+I_{M}⊗B_{0}K_{03}-\overset{―}{\alpha }I_{M}⊗B_{0}K_{01}1_{n\times n}$ and ${\tilde{A}}_{N}=I_{N}⊗A+ℒ⊗BK_{1}+\overset{―}{BK_{2}}+I_{N}⊗BK_{3}-\overset{―}{\nu }I_{N}⊗BK_{1}1_{n\times n}$. Therefore, the following relation is obtained:


$$\begin{array}{cc} \dot{\tilde{E}}(t) & ⪯(\begin{array}{cc} \begin{array}{c} {\tilde{A}}_{M0} \end{array} & 0\\ -B_{K_{2}} & {\tilde{A}}_{N} \end{array})\tilde{E}(t)+(\begin{array}{cc} I_{M}⊗{\bar{A}}_{0} & 0\\ 0 & \begin{array}{c} I_{N}⊗\bar{A} \end{array} \end{array})(\begin{array}{c} X_{0}^{*}\\ X^{*} \end{array}). \end{array}$$ (24)

Choose a CLF $V(\tilde{E}(t))={\tilde{E}}^{⊤}(t)\vartheta =E_{0}^{⊤}(t)(1_{M}⊗v_{0})+E^{⊤}(t)(1_{N}⊗v)$. Using (24) yields that


$$\begin{array}{cc} \dot{V}(t) & \leq (\begin{array}{cc} E_{0}^{⊤}(t) & E^{⊤}(t) \end{array})(\begin{array}{cc} \Xi_{1}^{⊤} & -B_{K_{2}}^{⊤}\\ 0 & \Xi_{2}^{⊤} \end{array})(\begin{array}{c} 1_{M}⊗v_{0}\\ 1_{N}⊗v \end{array})\\ & \;+(\begin{array}{cc} X_{0}^{*⊤}(t) & X^{*⊤}(t) \end{array})(\begin{array}{cc} \Xi_{3}^{⊤} & 0\\ 0 & \Xi_{4}^{⊤} \end{array})(\begin{array}{c} 1_{M}⊗v_{0}\\ 1_{N}⊗v \end{array})\\ & =E_{0}^{⊤}(t)(\Xi_{1}^{⊤}(1_{M}⊗v_{0})-B_{K_{2}}^{⊤}(1_{N}⊗v))+E^{⊤}(t)\\ & \;\times (\Xi_{2}^{⊤}(1_{N}⊗v))+(X_{0}^{*}{)}^{⊤}\Xi_{3}^{⊤}(1_{M}⊗v_{0})+(X^{*}{)}^{⊤}\Xi_{4}^{⊤}(1_{N}⊗v), \end{array}$$ (25)

where


$$\begin{array}{c} \begin{array}{cc} & \;\;\Xi_{1}^{⊤}(1_{M}⊗v_{0})-B_{K_{2}}^{⊤}(1_{N}⊗v)\\ & \;=[\Pi_{11}^{⊤},\cdots ,\Pi_{1Q}^{⊤},\Pi_{2(Q+1)}^{⊤},\cdots ,\Pi_{2M}^{⊤}{]}^{⊤},\\ \;\Pi_{1k} & \;=A_{0}v_{0}+\sum_{s\in {ℳ}_{k}}b_{ks}K_{01}^{⊤}B_{0}^{⊤}v_{0}-\sum_{s\in {ℳ}_{k}}b_{sk}K_{01}^{⊤}B_{0}^{⊤}v_{0}\\ & \;\;+K_{02}^{⊤}B_{0}^{⊤}v_{0}+K_{03}^{⊤}B_{0}^{⊤}v_{0}-\overset{―}{\alpha }1_{n\times n}K_{01}^{⊤}B_{0}^{⊤}v_{0}-K_{2}^{⊤}B^{⊤}v,\\ \;\Pi_{2k} & \;=A_{0}v_{0}+\sum_{s\in {ℳ}_{k}}b_{ks}K_{01}^{⊤}B_{0}^{⊤}v_{0}-\sum_{s\in {ℳ}_{k}}b_{sk}K_{01}^{⊤}\\ & \;\;\times B_{0}^{⊤}v_{0}+K_{03}^{⊤}B_{0}^{⊤}v_{0}-\overset{―}{\alpha }1_{n\times n}K_{01}^{⊤}B_{0}^{⊤}v_{0},\\ \Xi_{2}^{⊤}(1_{N}⊗v) & \;=[\Lambda_{11}^{⊤},\cdots ,\Lambda_{1M}^{⊤},\Lambda_{1(M+1)}^{⊤},\cdots ,\Lambda_{1N}^{⊤}{]}^{⊤},\\ \;\Lambda_{1i} & \;=Av+\sum_{j\in {𝒩}_{i}}a_{ij}K_{1}^{⊤}B^{⊤}v-\sum_{j\in {𝒩}_{i}}a_{ji}K_{1}^{⊤}B^{⊤}v\\ & \;\;+K_{2}^{⊤}B^{⊤}v+K_{3}^{⊤}B^{⊤}v-\overset{―}{\nu }1_{n\times n}K_{1}^{⊤}B^{⊤}v,\\ \;\Lambda_{2i} & \;=Av+\sum_{j\in {𝒩}_{i}}a_{ij}K_{1}^{⊤}B^{⊤}v-\sum_{j\in {𝒩}_{i}}a_{ji}K_{1}^{⊤}B^{⊤}v\\ & \;\;+K_{3}^{⊤}B^{⊤}v-\overset{―}{\nu }1_{n\times n}K_{1}^{⊤}B^{⊤}v, \end{array} \end{array}$$

$$\begin{array}{c} \begin{array}{cc} & \;\;(X_{0}^{*}{)}^{⊤}\Xi_{3}^{⊤}(1_{M}⊗v_{0})+(X^{*}{)}^{⊤}\Xi_{4}^{⊤}(1_{N}⊗v)\\ & \;=(X^{*}{)}^{⊤}(\Phi ⊗A_{0}^{⊤}v_{0}+\Phi ⊗F^{⊤}B_{0}^{⊤}v_{0}+\Phi ⊗G^{⊤}B_{0}^{⊤}v_{0}\\ & \;\;+I_{N}⊗A^{⊤}v+I_{N}⊗K_{3}^{⊤}B^{⊤}v+I_{N}⊗H^{⊤}B^{⊤}v),\\ \;\Xi_{1} & \;=I_{M}⊗A_{0}+{ℒ}_{0}⊗B_{0}K_{01}+\overset{―}{B_{0}K_{02}}+B_{0}K_{03}-\overset{―}{\alpha }I_{M}⊗B_{0}K_{01}1_{n\times n},\\ \;\Xi_{2} & \;=I_{N}⊗A+ℒ⊗BK_{1}+\overset{―}{BK_{2}}+BK_{3}-\overset{―}{\nu }I_{N}⊗BK_{1}1_{n\times n},\\ \;\Xi_{3} & \;=I_{M}⊗(A_{0}+B_{0}K_{03}+B_{0}G),\\ \;\Xi_{4} & \;=I_{N}⊗(A+BK_{3}BH). \end{array} \end{array}$$

Based on (11l) and ((12), one can get


$$\begin{array}{c} K_{01}⪯\frac{1_{r}\beta^{⊤}}{1_{r}^{⊤}B_{0}^{⊤}v_{0}},K_{02}⪯\frac{1_{r}\gamma^{⊤}}{1_{r}^{⊤}B_{0}^{⊤}v_{0}},K_{03}⪯\frac{1_{r}\eta^{⊤}}{1_{r}^{⊤}B_{0}^{⊤}v_{0}},G⪯\frac{1_{r}\sigma^{⊤}}{1_{r}^{⊤}B_{0}^{⊤}v_{0}},\\ K_{1}⪯\frac{1_{r}\delta^{⊤}}{1_{r}^{⊤}B^{⊤}v},K_{2}⪯\frac{1_{r}\zeta^{⊤}}{1_{r}^{⊤}B^{⊤}v},K_{3}⪯\frac{1_{r}\theta^{⊤}}{1_{r}^{⊤}B^{⊤}v},H⪯\frac{1_{r}\varsigma^{⊤}}{1_{r}^{⊤}B_{0}^{⊤}v_{0}}. \end{array}$$

Furthermore, we have


$$\begin{array}{c} \begin{array}{cc} \Pi_{1k} & \;⪯A_{0}v_{0}+\sum_{s\in {ℳ}_{k}}b_{ks}\beta -\sum_{s\in {ℳ}_{k}}b_{sk}\beta +\gamma \\ & \;\;+\eta -\overset{―}{\alpha }1_{n\times n}\beta -\zeta ,\\ \Pi_{2k} & \;⪯A_{0}v_{0}+\sum_{s\in {ℳ}_{k}}b_{ks}\beta -\sum_{s\in {ℳ}_{k}}b_{sk}\beta +\eta \\ & \;\;-\overset{―}{\alpha }1_{n\times n}\beta ,\\ \Lambda_{1i} & \;⪯Av+\sum_{j\in {𝒩}_{i}}a_{ij}\delta -\sum_{j\in {𝒩}_{i}}a_{ji}\delta +\zeta +\theta -\overset{―}{\nu }1_{n\times n}\delta , \end{array} \end{array}$$

$$\begin{array}{c} \begin{array}{cc} \Lambda_{2i} & \;⪯Av+\sum_{j\in {𝒩}_{i}}a_{ij}\delta -\sum_{j\in {𝒩}_{i}}a_{ji}\delta +\theta -\overset{―}{\nu }1_{n\times n}\delta ,\\ & \;\;(X_{0}^{*}{)}^{⊤}\Xi_{3}^{⊤}(1_{M}⊗v_{0})+(X^{*}{)}^{⊤}\Xi_{4}^{⊤}(1_{N}⊗v)\\ & \;\leq (X^{*}{)}^{⊤}(\Phi ⊗A_{0}^{⊤}v_{0}+\Phi ⊗\eta +\Phi ⊗\sigma +1_{N}⊗A^{⊤}v\\ & \;\;+1_{N}⊗\theta +1_{N}⊗\varsigma ). \end{array} \end{array}$$

By (11g-11k), the condition (25) can be transformed into $\dot{V}(t)<-\omega_{1}{\tilde{E}}^{⊤}(t)\vartheta +\omega_{2}(X^{*}{)}^{⊤}\times (1_{N}⊗v_{a})$. It derives that $\lim_{t\to \infty }\|x_{0k}(t)-x_{0}^{*}{\|}_{1}<℧$, where $℧=\frac{\omega_{2}}{\hbar \omega_{1}}(X^{*}{)}^{⊤}(1_{N}⊗v_{a})$, ħ $\begin{array}{c} =\min_{\ell \in \{1,2,\cdots ,n\}}\{v_{0\ell }\} \end{array}$, and $v_{0\ell }$ is the component of *v*_0_. Therefore, the practical consensus of the system (20) is guaranteed by Definition 2. ∎.

**Remark 2** Theorem 1 provides linear programming conditions for matrix gain design, positivity, and consensus. These conditions can be solved using the linear programming toolbox in MATLAB. Since the multi-leader and multi-follower agents considered in this paper are homogeneous, taking Theorem 1 as an example, it can be observed that the computational complexity of the proposed conditions depends solely on the dimension of the agent system. This implies that, theoretically, the proposed linear programming conditions are computable regardless of the system’s scale. However, when the system is heterogeneous, the computational complexity of the conditions becomes related to both the system dimension and the system size. In such cases, when the system size is exceptionally large (e.g., exceeding 1000 nodes), computing the linear programming conditions becomes infeasible due to computational device limitations.

**Remark 3** To solve for the gain matrix, Theorem 1 introduces some unknown decision variables. By analyzing these decision variables and the number of inequality constraints, the computational complexity of Theorem 1 can be determined. Calculating the number of vectors in the gain matrix and Lyapunov function yields a total number of decision variables of $\bar{n}=11n+8nr$. Similarly, the total number of inequality constraints is $\bar{m}=19$. Then, combining the discretization of the state space with the theoretical complexity of the interior-point method, the computational complexity for solving Theorem 1 is determined to be $O(\bar{m}{\bar{N}}^{3.5\bar{n}})$, where $\bar{N}$ is the number of discretization points. Although this complexity increases exponentially with the system dimension, the matrix A in the MATLAB function *linprog* possesses a sparse structure. This significantly enhances the efficiency of its solution compared to linear matrix inequality methods.

**Remark 4** For non-positive MASs, multiple Lyapunov-Krasovskii functionals [17], candidate Lyapunov function [32] and others are used for consensus analysis. However, these Lyapunov functions are overly complex for the consensus analysis of MAS. Fortunately, the CLF offers a more concise approach for analyzing the stability of positive systems compared to the aforementioned methods. At the same time, LP is computationally simpler than linear matrix inequality. Therefore, in Theorem 1, CLF is used to address the consensus of PMASs and LP is utilized to solve the corresponding conditions.

**Remark 5** The literature [25] investigated asymptotic consensus. It means that the states of all agents converge to a common value. This consensus will keep all agents at the same trajectory. However, the system may be exposed to various uncertainties and variations. The overall performance of the system may be severely affected when one or more agents are broken down. The behaviour of the failed agents may be different from other agents and thus the system will not be able to achieve the desired consensus state. Therefore, in this paper, practical consensus is achieved by adding $x_{0}^{*}$ to the controller and all agent states will be in a region. This approach ensures that the failure of any agent does not affect the overall system, thereby enhancing the system’s stability.

**Remark 6** To prove that Zeno’s behavior will not occur, taking the event-triggered mechanism of the leader in Section 3.1 as an example, the following procedure gives the minimum time interval between two consecutive samples. Let $\Phi (t)=\frac{\|\phi (t){\|}_{2}}{\|X_{0}(t){\|}_{2}}$. Assume there exist positive constants $\Delta_{1}$, $\Delta_{2}$, and $\Delta_{3}$ such that $\Delta_{3}\|X_{0}(t){\|}_{2}\leq \|E_{0}(t){\|}_{2}\leq \Delta_{1}\|X_{0}(t){\|}_{2}$, $\|X_{0}^{*}{\|}_{2}\leq \Delta_{2}\|X_{0}(t){\|}_{2}$, hold. Then, we can obtain


$$\begin{array}{cc} \dot{\Phi }(t) & =\frac{\phi^{T}(t)\dot{\phi }(t)}{\|\phi (t){\|}_{2}\|X_{0}(t){\|}_{2}}-\frac{\left\|\phi (t){\|}_{2}X_{0}^{T}(t)X_{0}(t\right)}{\|X_{0}(t){\|}_{2}^{3}}\\ & \leq \frac{\|\dot{\phi }(t){\|}_{2}}{\|X_{0}(t){\|}_{2}}+\frac{\|\phi (t){\|}_{2}\|{\dot{X}}_{0}(t){\|}_{2}}{\|X_{0}(t){\|}_{2}^{2}}\\ & \leq \frac{\|{\dot{Z}}_{0}(t){\|}_{2}}{\|X_{0}(t){\|}_{2}}+\frac{\overset{―}{\alpha }\sqrt{nM}\|E_{0}(t){\|}_{2}\|{\dot{X}}_{0}(t){\|}_{2}}{\|X_{0}(t){\|}_{2}^{2}}\\ & \leq \frac{(\|{ℒ}_{0}\|+\overset{―}{\alpha }\sqrt{nM}\Delta_{1})\|{\dot{X}}_{0}(t){\|}_{2}}{\|X_{0}(t){\|}_{2}}\\ & \leq \Omega_{1}(\Omega_{2}+\Omega_{3}+\Omega_{4}), \end{array}$$

where $X_{0}(t)=(x_{01}^{T}(t),\cdots ,x_{0M}^{T}(t))$, $\Omega_{1}=\|{ℒ}_{0}{\|}_{2}+\overset{―}{\alpha }\sqrt{nM}\Delta_{1}$, $\Omega_{2}=\|I_{M}⊗A_{0}+{ℒ}_{0}⊗B_{0}K_{01}+I_{M}⊗B_{0}K_{03}{\|}_{2}$, $\Omega_{3}=\overset{―}{\alpha }\sqrt{nM}\Delta_{1})$ $\|I_{M}⊗B_{0}K_{01}{\|}_{2}+\Delta_{1}\|I_{M}⊗B_{0}K_{02}{\|}_{2}$, $\Omega_{4}=\Delta_{2}\|I_{M}⊗B_{0}G{\|}_{2}$, and ${\dot{X}}_{0}(t)=(I_{M}⊗A_{0}+{ℒ}_{0}⊗B_{0}K_{01}+I_{M}⊗B_{0}K_{03})$ $X_{0}(t)+(I_{M}⊗B_{0}K_{01})\phi (t)+(I_{M}⊗B_{0}K_{02})E_{0}(t)+(I_{M}⊗B_{0}G)X_{0}^{*}$. Denote $\Omega =\Omega_{1}(\Omega_{2}+\Omega_{3}+\Omega_{4})$, then $\dot{\Phi }(t)<\Omega$. By employing the comparison principle, one can get $\Phi (t)-\Phi (t_{p})\leq \Omega (t-t_{p})$, where *t*_*p*_ is the *p*th event-triggered time instant and satisfies $\Phi (t_{p}=0$. Based on condition (9), it can be deduced that $\overset{―}{\alpha }\|E_{0}(t){\|}_{1}<\|\phi (t){\|}_{1}<\sqrt{nM}\|\phi (t){\|}_{2}<\sqrt{nM}\Omega (t-t_{p})\|X_{0}(t){\|}_{2}$. Thus, $t-t_{p}>\frac{\overset{―}{\alpha }\|E_{0}(t){\|}_{2}}{\sqrt{nM}\Omega \|X_{0}(t){\|}_{2}}>\frac{\overset{―}{\alpha }\Delta_{3}}{\sqrt{nM}\Omega }$. This means that Zeno’s behavior can be avoided.

### 3.2 Observer-based consensus

Design the pinning observer ${\hat{x}}_{0k}(t)$ for leaders as


$$\begin{array}{cc} {\dot{\hat{x}}}_{0k}(t) & =A_{0}{\hat{x}}_{0k}(t)+B_{0}u_{0k}(t)+L_{0}({\hat{y}}_{0k}(t)-y_{0k}(t_{h}^{k}))+F_{0}y_{0k}(t),\;k=1,2,\ldots ,Q,\\ {\dot{\hat{y}}}_{0k}(t) & =C_{0}{\hat{x}}_{0k}(t), \end{array}$$ (26)

where ${\hat{y}}_{i}(t)\in {𝐑}^{q}$ is the observer output, *L*_0_ and *F*_0_ are the gain matrix to be determined, and $t_{h}^{k}$, h=1,2,⋯, is the event-triggered time sequence of the *i*th observer. Based on the observer, the corresponding controller is designed as:


$$\begin{array}{cc} u_{0k}(t) & =K_{01}{\hat{z}}_{0k}^{a}(t_{p}^{k})+K_{02}({\hat{x}}_{0k}(t)-x_{0}^{*})+K_{03}{\hat{x}}_{0k}(t)\\ & \;+K_{06}({\hat{y}}_{0k}(t)-y_{0k}(t))+Gx_{0}^{*},\;k=1,2,\cdots ,Q,\\ u_{0k}(t) & =K_{04}z_{0k}^{b}(t_{p}^{k})+K_{05}x_{0k}(t)+Gx_{0}^{*},\;k=Q+1,Q+2,\cdots ,M, \end{array}$$ (27)

where $t_{p}^{k}$ is the sequence of triggers for leader *k* and *K*_01_, *K*_02_, *K*_03_, *K*_04_, *K*_05_, *K*_06_, and *G* are gain matrices. The relative measurements ${\hat{z}}_{i}^{a}(t)$ and $z_{i}^{b}(t)$ are defined as


$$\begin{array}{c} \begin{array}{cc} {\hat{z}}_{0k}^{a}(t) & \;=\sum_{s\in {ℳ}_{k}}a_{ks}({\hat{x}}_{0k}(t)-{\hat{x}}_{0s}(t))+\sum_{l\in {ℳ}_{k}}a_{kl}\\ & \;\;\times ({\hat{x}}_{0k}(t)-x_{0l}(t)),\;k=1,2,\cdots ,Q,\\ z_{0k}^{b}(t) & \;=\sum_{s\in {ℳ}_{k}}b_{ks}(x_{0k}(t)-{\hat{x}}_{0s}(t))+\sum_{l\in {ℳ}_{k}}b_{kl}\\ & \;\;\times (x_{0k}(t)-x_{0l}(t)),\;k=Q+1,Q+2,\cdots ,M, \end{array} \end{array}$$ (28)

where s=1,2,⋯,Q, l=Q+1,Q+2,⋯,M and ${ℳ}_{k}$ is the in-neighbor set of agent *k*. For pinned agents, if the *i*th agent can receive the information from the *j*th agent, *a*_*ks*_ > 0 or *a*_*kl*_ > 0; otherwise, *b*_*ks*_ = 0 or *b*_*kl*_ = 0. For non-pinned agents, it has the same property. The pinning observer ${\hat{x}}_{i}(t)$ for followers can be given by


$$\begin{array}{c} {\dot{\hat{x}}}_{i}(t)=A{\hat{x}}_{i}(t)+Bu_{i}(t)+L({\hat{y}}_{i}(t)-y_{i}(t_{d}^{i}))+Fy_{i}(t),\;i=1,2,\cdots ,M,\\ {\dot{\hat{y}}}_{i}(t)=C{\hat{x}}_{i}(t). \end{array}$$ (29)

The pinning controller of the follower system are


$$\begin{array}{c} u_{i}(t)=K_{1}{\hat{z}}_{i}^{a}(t_{\sigma }^{i})+K_{2}\sum_{k=1}^{M}c_{ik}({\hat{x}}_{i}(t)-x_{0k}(t))\\ \;\;\;+K_{3}{\hat{x}}_{i}(t)+K_{6}({\hat{y}}_{i}(t)-y_{i}(t))+Hx_{0}^{*},i=1,2,\cdots ,M,\\ u_{i}(t)=K_{4}z_{i}^{b}(t_{\sigma }^{i})+K_{5}x_{i}(t)+Hx_{0}^{*},\;i=M+1,M+2,\cdots ,N, \end{array}$$ (30)

where $t_{\sigma }^{i}$ is the sequence of communication triggers for follower *i*. If there are information interactions between leaders and followers, *c*_*ik*_ = 1; otherwise *c*_*ik*_ = 0. *K*_1_, *K*_2_, *K*_3_, *K*_4_, *K*_5_, *K*_6_, and *H* are gain matrices. The state thresholds $z_{i}^{a}(t)$ and $z_{i}^{b}(t)$ are defined as


$$\begin{array}{c} \begin{array}{cc} {\hat{z}}_{i}^{a}(t) & \;=\sum_{j\in {𝒩}_{i}}a_{ij}({\hat{x}}_{i}(t)-{\hat{x}}_{j}(t))+\sum_{o\in {𝒩}_{i}}a_{io}\\ & \;\;\times ({\hat{x}}_{i}(t)-x_{o}(t)),\;i=1,2,\cdots ,M,\\ z_{i}^{b}(t) & \;=\sum_{j\in {𝒩}_{i}}b_{ij}(x_{i}(t)-{\hat{x}}_{j}(t))+\sum_{o\in {𝒩}_{i}}b_{io}\\ & \;\;\times (x_{i}(t)-x_{o}(t)),\;i=M+1,M+2,\cdots ,N, \end{array} \end{array}$$ (31)

where ${𝒩}_{i}$ is the in-neighbor set of agent *i*, ${\hat{z}}_{i}^{a}(t)$ and $z_{i}^{b}(t)$ have the same properties as ${\hat{z}}_{0k}^{a}(t)$ and $z_{0k}^{b}(t)$. Define the sampling error $\Phi_{k}(t)$ and $\Psi_{i}(t)$ by


$$\begin{array}{c} \Phi_{k}(t)=y_{0k}(t_{h}^{k})-y_{0k}(t),\;\Psi_{i}(t)=y_{i}(t_{d}^{i})-y_{i}(t). \end{array}$$ (32)

The event-triggered conditions can be constructed as


$$\begin{array}{c} \|\Phi_{k}(t){\|}_{1}>\pi_{k}(t)\|y_{0k}(t){\|}_{1},\;\|\Psi_{i}(t){\|}_{1}>\omega_{i}(t)\|y_{i}(t){\|}_{1}, \end{array}$$ (33)

where


$$\begin{array}{c} {\dot{\pi }}_{k}(t)=(\pi_{k}(t)-\Pi_{k})(\|\Phi_{k}(t){\|}_{1}-\overset{―}{\pi }\|y_{0k}(t){\|}_{1}),\\ {\dot{\omega }}_{i}(t)=(\omega_{i}(t)-{ℶ}_{i})(\|\Psi_{i}(t){\|}_{1}-\overset{―}{\omega }\|y_{i}(t){\|}_{1}), \end{array}$$ (34)

$0<\Pi_{k}<\pi_{k}(t_{0})<\overset{―}{\pi }$, $0<{ℶ}_{i}<\omega_{i}(t_{0})<\overset{―}{\omega }$. $\pi_{k}(t_{0})$ and $\omega_{i}(t_{0})$ are initial conditions. $\Pi_{k}$, ${ℶ}_{i}$, ${\overset{―}{\pi }}_{k}$, and ${\overset{―}{\omega }}_{i}$ are known constants.

Next, the observer-based pinning control and multi-adaptive event-triggered consensus are achieved for PMASs.

**Theorem 2** If there exist constants $\epsilon_{1}>0$, $\epsilon_{2}>0$, $\epsilon_{3}>0$, $\epsilon_{4}>0$, $\varpi_{1}>0$, $\varpi_{2}>0$, ${\mathbb{R}}^{n}$ vectors $v_{0}≻0$, $v_{1}≻0$, $v_{2}≻0$, $v_{3}≻0$, $v_{a}≻0$, $\beta_{ı}≺0$, $\gamma_{ı}≺0$, $\eta_{ı}≺0$, $\kappa_{ı}≺0$, $\xi_{ı}≻0$, $\psi_{ı}≻0$, $\pi_{ı}≻0$, $\delta_{ı}≺0$, $\zeta_{ı}≺0$, $\theta_{ı}≺0$, $o_{ı}≺0$, $\varrho_{ı}≺0$, $\chi_{ı}≻0$, $\rho_{ı}≻0$, β≺0, γ≺0, η≺0, κ≺0, ξ≻0, ψ≻0, π≻0, δ≺0, ζ≺0, θ≺0, o≺0, ϱ≺0, χ≺0, ρ≻0, and ${\mathbb{R}}^{q}$ vectors $\sigma_{ı}≻0$, $\varsigma_{ı}≺0$, $\tau_{ı}≻0$, $\varepsilon_{ı}≺0$, σ≻0, ς≺0, τ≻0, ε≺0 such that inequalities


$$\begin{array}{c} 1_{r}^{⊤}B_{0}^{⊤}v_{0}A_{0}+\sum_{s\in {ℳ}_{k}}b_{ks}B_{0}\sum_{ı=1}^{r}1_{r}^{\left(ı\right)}\beta_{ı}^{⊤}+B_{0}\sum_{ı=1}^{r}1_{r}^{\left(ı\right)}\gamma_{ı}^{⊤}\\ +B_{0}\sum_{ı=1}^{r}1_{r}^{\left(ı\right)}\eta_{ı}^{⊤}+\overset{―}{\alpha }B_{0}\sum_{ı=1}^{r}1_{r}^{\left(ı\right)}\beta_{ı}^{⊤}1_{n\times n}+\epsilon_{1}I⪰0,\;k=1,2,\cdots ,Q, \end{array}$$ (35a)

$$\begin{array}{c} 1_{r}^{⊤}B_{0}^{⊤}v_{0}A_{0}+\sum_{s\in {ℳ}_{k}}b_{kl}B_{0}\sum_{ı=1}^{r}1_{r}^{\left(ı\right)}\kappa_{ı}^{⊤}+B_{0}\sum_{ı=1}^{r}1_{r}^{\left(ı\right)}\xi_{ı}^{⊤}\\ +\overset{―}{\alpha }B_{0}\sum_{ı=1}^{r}1_{r}^{\left(ı\right)}\kappa_{ı}^{⊤}1_{n\times n}+\epsilon_{1}I⪰0,k=Q+1,Q+2,\cdots ,M, \end{array}$$ (35b)

$$\begin{array}{c} 1_{n}^{⊤}v_{1}A_{0}+\sum_{ı=1}^{n}1_{n}^{\left(ı\right)}\varsigma_{ı}^{⊤}C_{0}+\epsilon_{2}I⪰0, \end{array}$$ (35c)

$$\begin{array}{c} 1_{n}^{⊤}v_{3}A+\sum_{ı=1}^{n}1_{n}^{\left(ı\right)}\varepsilon_{ı}^{⊤}C+\epsilon_{3}I⪰0, \end{array}$$ (35d)

$$\begin{array}{c} 1_{r}^{⊤}B^{⊤}v_{2}A+\sum_{j\in {𝒩}_{i}}a_{ij}B\sum_{ı=1}^{r}1_{r}^{\left(ı\right)}\delta_{ı}^{⊤}+B\sum_{ı=1}^{r}1_{r}^{\left(ı\right)}\zeta_{ı}^{⊤}\\ +B\sum_{ı=1}^{r}1_{r}^{\left(ı\right)}\theta_{ı}^{⊤}+\overset{―}{\nu }B\sum_{ı=1}^{r}1_{r}^{\left(ı\right)}\delta_{ı}^{⊤}1_{n\times n}+\epsilon_{4}I⪰0,k=1,2,\cdots ,M, \end{array}$$ (35e)

$$\begin{array}{c} 1_{r}^{⊤}B^{⊤}v_{2}A+\sum_{o\in {𝒩}_{i}}a_{io}B\sum_{ı=1}^{r}1_{r}^{\left(ı\right)}o_{ı}^{⊤}+B\sum_{ı=1}^{r}1_{r}^{\left(ı\right)}\varrho_{ı}^{⊤}\\ +\overset{―}{\nu }B\sum_{ı=1}^{r}1_{r}^{\left(ı\right)}o_{ı}^{⊤}1_{n\times n}+\epsilon_{4}I⪰0,k=M+1,M+2,\cdots ,N, \end{array}$$ (35f)

$$\begin{array}{c} \sum_{s\in {ℳ}_{k}}b_{ks}B_{0}\sum_{ı=1}^{r}1_{r}^{\left(ı\right)}\beta_{ı}^{⊤}+B_{0}\sum_{ı=1}^{r}1_{r}^{\left(ı\right)}\gamma_{ı}^{⊤}\\ +B_{0}\sum_{ı=1}^{r}1_{r}^{\left(ı\right)}\psi_{ı}^{⊤}C_{0}+B_{0}\sum_{ı=1}^{r}1_{r}^{\left(ı\right)}\eta_{ı}^{⊤}⪰0, \end{array}$$ (35g)

$$\begin{array}{c} \sum_{j\in {𝒩}_{i}}a_{ij}B\sum_{ı=1}^{r}1_{r}^{\left(ı\right)}\delta_{ı}^{⊤}+B\sum_{ı=1}^{r}1_{r}^{\left(ı\right)}\zeta_{ı}^{⊤}\\ +B\sum_{ı=1}^{r}1_{r}^{\left(ı\right)}\chi_{ı}^{⊤}C+B\sum_{ı=1}^{r}1_{r}^{\left(ı\right)}\theta_{ı}^{⊤}⪰0, \end{array}$$ (35h)

$$\begin{array}{c} \sum_{ı=1}^{n}1_{n}^{\left(ı\right)}\sigma_{ı}^{⊤}C_{0}+{\overset{―}{\pi }\sum }_{ı=1}^{n}1_{n}^{\left(ı\right)}\varsigma_{ı}^{⊤}1_{q\times q}C_{0}⪰0, \end{array}$$ (35i)

$$\begin{array}{c} \sum_{ı=1}^{n}1_{n}^{\left(ı\right)}\tau_{ı}^{⊤}C+{\overset{―}{\omega }\sum }_{ı=1}^{n}1_{n}^{\left(ı\right)}\varepsilon_{ı}^{⊤}1_{q\times q}C⪰0, \end{array}$$ (35j)

$$\begin{array}{c} (1_{r}^{⊤}B_{0}^{⊤}v_{0}A_{0}+B_{0}\sum_{ı=1}^{r}1_{r}^{\left(ı\right)}\eta_{ı}^{⊤}B_{0}\sum_{ı=1}^{r}1_{r}^{\left(ı\right)}\pi_{ı}^{⊤})x_{0}^{*}⪰0, \end{array}$$ (35k)

$$\begin{array}{c} (1_{r}^{⊤}B_{0}^{⊤}v_{0}A_{0}+B_{0}\sum_{ı=1}^{r}1_{r}^{\left(ı\right)}\xi_{ı}^{⊤}+B_{0}\sum_{ı=1}^{r}1_{r}^{\left(ı\right)}\pi_{ı}^{⊤})x_{0}^{*}⪰0, \end{array}$$ (35l)

$$\begin{array}{c} (1_{r}^{⊤}B^{⊤}v_{2}A+B\sum_{ı=1}^{r}1_{r}^{\left(ı\right)}\theta_{ı}^{⊤}+B\sum_{ı=1}^{r}1_{r}^{\left(ı\right)}\rho_{ı}^{⊤})x_{0}^{*}⪰0, \end{array}$$ (35m)

$$\begin{array}{c} (1_{r}^{⊤}B^{⊤}v_{2}A+B\sum_{ı=1}^{r}1_{r}^{\left(ı\right)}\varrho_{ı}^{⊤}+B\sum_{ı=1}^{r}1_{r}^{\left(ı\right)}\rho_{ı}^{⊤})x_{0}^{*}⪰0, \end{array}$$ (35n)

$$\begin{array}{c} A_{0}^{⊤}v_{0}+\sum_{s\in {ℳ}_{k}}a_{ks}\beta -\sum_{s\in {ℳ}_{k}}a_{sk}\beta -\sum_{l\in {ℳ}_{k}}b_{lk}\kappa +\gamma \\ +\eta -\overset{―}{\alpha }1_{n\times n}\beta +C_{0}^{⊤}\sigma -\overset{―}{\pi }C_{0}^{⊤}1_{q\times q}\varsigma -\zeta +\varpi_{1}v_{0}≺0, \end{array}$$ (35o)

$$\begin{array}{c} A_{0}^{⊤}v_{0}+\sum_{l\in {ℳ}_{k}}b_{kl}\kappa -\sum_{l\in {ℳ}_{k}}b_{lk}\kappa -\sum_{s\in {ℳ}_{k}}a_{sk}\beta \\ +\xi -\overset{―}{\alpha }1_{n\times n}\kappa +\varpi_{1}v_{0}≺0, \end{array}$$ (35p)

$$\begin{array}{c} \sum_{s\in {ℳ}_{k}}a_{ks}\beta -\sum_{s\in {ℳ}_{k}}a_{sk}\beta -\sum_{l\in {ℳ}_{k}}b_{lk}\kappa +\gamma \\ +\psi +\eta +A_{0}^{⊤}v_{1}+C_{0}^{⊤}\varsigma +\varpi_{1}v_{1}≺0, \end{array}$$ (35q)

$$\begin{array}{c} A^{⊤}v_{2}+\sum_{j\in {𝒩}_{i}}a_{ij}\delta -\sum_{j\in {𝒩}_{i}}a_{ji}\delta -\sum_{o\in {𝒩}_{i}}b_{oi}o+\zeta \\ +\theta -\overset{―}{\nu }1_{n\times n}\delta +C^{⊤}\tau -\overset{―}{\omega }C^{⊤}1_{q\times q}\varepsilon +\varpi_{1}v_{2}≺0, \end{array}$$ (35r)

$$\begin{array}{c} A^{⊤}v_{2}+\sum_{o\in {𝒩}_{i}}b_{io}o-\sum_{o\in {𝒩}_{i}}b_{oi}o-\sum_{j\in {𝒩}_{i}}a_{ji}\delta \\ +\varrho -\overset{―}{\nu }1_{n\times n}o+\varpi_{1}v_{2}≺0, \end{array}$$ (35s)

$$\begin{array}{c} \sum_{j\in {𝒩}_{1}}a_{1j}\delta -\sum_{j\in {𝒩}_{1}}a_{j1}\delta -\sum_{o\in {𝒩}_{1}}b_{o1}o+\zeta +\chi \\ +\theta +A^{⊤}v_{3}+C^{⊤}\varepsilon +\varpi_{1}v_{3}≺0, \end{array}$$ (35t)

$$\begin{array}{c} 1_{ℜ}⊗A_{0}^{⊤}v_{0}+1_{ℵ}⊗\eta +1_{℧}⊗\xi +1_{ℜ}⊗\pi +1_{ℵ}⊗C_{0}^{⊤}\sigma \\ -\overset{―}{\pi }1_{ℵ}⊗C_{0}^{⊤}1_{q\times q}\varsigma +1_{N}⊗A^{⊤}v_{2}+1_{ℜ}⊗\theta +1_{ℷ}⊗\varrho +1_{N}⊗\rho \\ +1_{ℜ}⊗C^{⊤}\tau -\overset{―}{\omega }1_{ℜ}⊗C^{⊤}1_{q\times q}\varepsilon -\varpi_{2}(1_{N}⊗v_{a})≺0, \end{array}$$ (35u)

$$\begin{array}{c} \beta_{ı}⪯\beta ,\gamma_{ı}⪯\gamma ,\eta_{ı}⪯\eta ,\kappa_{ı}⪯\kappa ,\xi_{ı}⪯\xi ,\psi_{ı}⪯\psi ,\pi_{ı}⪯\pi ,\delta_{ı}⪯\delta ,\zeta_{ı}⪯\zeta ,\\ \theta_{ı}⪯\theta ,o_{ı}⪯o,\varrho_{ı}⪯\varrho ,\chi_{ı}⪯\chi ,\rho_{ı}⪯\rho ,\sigma_{ı}⪯\sigma ,\varsigma_{ı}⪯\varsigma ,\tau_{ı}⪯\tau ,\varepsilon_{ı}⪯\varepsilon , \end{array}$$ (35v)

hold for ı=1,2,⋯,r, then under the pinned observer (26), (28), the leader’s control protocol (27) and the followers’ control protocol (30) with


$$\begin{array}{c} K_{01}=\frac{\sum_{ı=1}^{r}1_{r}^{\left(ı\right)}\beta_{ı}^{⊤}}{1_{r}^{⊤}B_{0}^{⊤}v_{0}},K_{02}=\frac{\sum_{ı=1}^{r}1_{r}^{\left(ı\right)}\gamma_{ı}^{⊤}}{1_{r}^{⊤}B_{0}^{⊤}v_{0}},K_{03}=\frac{\sum_{ı=1}^{r}1_{r}^{\left(ı\right)}\eta_{ı}^{⊤}}{1_{r}^{⊤}B_{0}^{⊤}v_{0}},K_{04}=\frac{\sum_{ı=1}^{r}1_{r}^{\left(ı\right)}\kappa_{ı}^{⊤}}{1_{r}^{⊤}B_{0}^{⊤}v_{0}},\\ K_{05}=\frac{\sum_{ı=1}^{r}1_{r}^{\left(ı\right)}\xi_{ı}^{⊤}}{1_{r}^{⊤}B_{0}^{⊤}v_{0}},K_{06}=\frac{\sum_{ı=1}^{r}1_{r}^{\left(ı\right)}\psi_{ı}^{⊤}}{1_{r}^{⊤}B_{0}^{⊤}v_{0}},G=\frac{\sum_{ı=1}^{r}1_{r}^{\left(ı\right)}\pi_{ı}^{⊤}}{1_{r}^{⊤}B_{0}^{⊤}v_{0}},K_{1}=\frac{\sum_{ı=1}^{r}1_{r}^{\left(ı\right)}\delta_{ı}^{⊤}}{1_{r}^{⊤}B^{⊤}v_{2}},\\ K_{2}=\frac{\sum_{ı=1}^{r}1_{r}^{\left(ı\right)}\zeta_{ı}^{⊤}}{1_{r}^{⊤}B^{⊤}v_{2}},K_{3}=\frac{\sum_{ı=1}^{r}1_{r}^{\left(ı\right)}\theta_{ı}^{⊤}}{1_{r}^{⊤}B^{⊤}v_{2}},K_{4}=\frac{\sum_{ı=1}^{r}1_{r}^{\left(ı\right)}o_{ı}^{⊤}}{1_{r}^{⊤}B^{⊤}v_{2}},K_{5}=\frac{\sum_{ı=1}^{r}1_{r}^{\left(ı\right)}\varrho_{ı}^{⊤}}{1_{r}^{⊤}B^{⊤}v_{1}},\\ K_{6}=\frac{\sum_{ı=1}^{r}1_{r}^{\left(ı\right)}\chi_{ı}^{⊤}}{1_{r}^{⊤}B^{⊤}v_{1}},H=\frac{\sum_{ı=1}^{r}1_{r}^{\left(ı\right)}\rho_{ı}^{⊤}}{1_{r}^{⊤}B^{⊤}v_{1}},F_{0}=\frac{\sum_{ı=1}^{n}1_{n}^{\left(ı\right)}\sigma_{ı}^{⊤}}{1_{n}^{⊤}v_{1}},L_{0}=\frac{\sum_{ı=1}^{n}1_{n}^{\left(ı\right)}\varsigma_{ı}^{⊤}}{1_{n}^{⊤}v_{1}},\\ F=\frac{\sum_{ı=1}^{n}1_{n}^{\left(ı\right)}\tau_{ı}^{⊤}}{1_{n}^{⊤}v_{3}},L=\frac{\sum_{ı=1}^{n}1_{n}^{\left(ı\right)}\varepsilon_{ı}^{⊤}}{1_{n}^{⊤}v_{3}}, \end{array}$$ (36)

the system (1) is positive and systems (1) and (2) reach the leader-following consensus, where $X_{0}^{*}=(\underset{M}{\underbrace{x_{0}^{*⊤},\ldots ,x_{0}^{*⊤}}}{)}^{⊤}$, $X^{*}=(\underset{N}{\underbrace{x_{0}^{*⊤},\ldots ,x_{0}^{*⊤}}}{)}^{⊤}$, $ℵ=(1_{Q}^{⊤},\underset{N-Q}{\underbrace{0,\ldots ,0}}{)}^{⊤}$, $℧=(\underset{Q}{\underbrace{0,\ldots ,0}},1_{M-Q}^{⊤},\underset{N-M}{\underbrace{0,\ldots ,0}}{)}^{⊤}$, $ℜ=(1_{M}^{⊤},\underset{N-M}{\underbrace{0,\ldots ,0}}{)}^{⊤}$, $ℷ=(\underset{M}{\underbrace{0,\ldots ,0}},1_{N-M}^{⊤}{)}^{⊤}$.

**Proof:** From (33), it yields that


$$\begin{array}{c} \|\Phi_{k}(t){\|}_{1}\leq \pi_{k}(t)\|y_{0k}(t){\|}_{1},\;\|\Psi_{i}(t){\|}_{1}\leq \omega_{i}(t)\|y_{i}(t){\|}_{1}. \end{array}$$ (37)

For $x_{0k}(t_{0})⪰0$, $x_{i}(t_{0})⪰0$, one has $y_{0k}(t_{0})⪰0$ and $y_{i}(t_{0})⪰0$. Then, the follwing inequalities


$$\begin{array}{c} -\overset{―}{\pi }1_{q\times q}y_{0k}(t_{0})⪯\Phi_{k}(t_{0})⪯\overset{―}{\pi }1_{q\times q}y_{0k}(t_{0}),\\ -\overset{―}{\omega }1_{q\times q}y_{i}(t_{0})⪯\Psi_{i}(t_{0})⪯\overset{―}{\omega }1_{q\times q}y_{i}(t_{0}) \end{array}$$ (38)

hold, where $\Phi_{k}(t_{0})$ and $\Psi_{i}(t_{0})$ are initial conditions. Define the following observer errors


$$\begin{array}{c} {\hat{e}}_{0k}(t)={\hat{x}}_{0k}(t)-x_{0k}(t) \end{array}$$ (39)

and


$$\begin{array}{c} {\hat{e}}_{i}(t)={\hat{x}}_{i}(t)-x_{i}(t). \end{array}$$ (40)

Then, in view of (2), (6), (8), (26)-(28), and (32), the follwing equations


$$\begin{array}{c} \begin{array}{cc} {\dot{e}}_{0k}(t) & \;=A_{0}e_{0k}(t)+B_{0}(K_{01}\sum_{s\in {ℳ}_{k}}a_{ks}({\hat{e}}_{0k}(t)+e_{0k}(t)-{\hat{e}}_{0s}(t)-e_{0s}(t))+K_{01}\sum_{l\in {ℳ}_{k}}\\ & \;\;\times a_{kl}({\hat{e}}_{0k}(t)+e_{0k}(t)-e_{0l}(t))+K_{01}\phi_{k}(t)+K_{02}({\hat{e}}_{0k}(t)+e_{0k}(t))+K_{03}({\hat{e}}_{0k}(t)\\ & \;\;+e_{0k}(t)))+K_{06}C_{0}{\hat{e}}_{0k}(t)+(A_{0}+B_{0}K_{03}+B_{0}G)x_{0}^{*},\\ {\dot{\hat{e}}}_{0k}(t) & \;=F_{0}C_{0}e_{0k}(t)+(A_{0}+L_{0}C_{0}){\hat{e}}_{0k}(t)-L_{0}\Phi_{k}(t)+F_{0}C_{0}x_{0}^{*} \end{array} \end{array}$$ (41)

are derived for k=1,2,⋯,Q. When k=Q+1,Q+2,⋯,M, it holds that


$$\begin{array}{c} \begin{array}{cc} {\dot{e}}_{0k}(t) & \;=A_{0}e_{0k}(t)+B_{0}(K_{04}\sum_{s\in {ℳ}_{k}}b_{ks}(e_{0k}(t)-e_{0s}(t)-{\hat{e}}_{0s}(t))+K_{04}\sum_{l\in {ℳ}_{k}}b_{kl}\\ & \;\;\times (e_{0k}(t)-e_{0l}(t))+K_{04}\phi_{k}(t)+K_{05}e_{0k}(t))+(A_{0}+B_{0}K_{05}+B_{0}G)x_{0}^{*}. \end{array} \end{array}$$ (42)

Similarly, for i=1,2,⋯,M, it can be obtained that


$$\begin{array}{c} \begin{array}{cc} {\dot{e}}_{i}(t) & \;=Ae_{i}(t)+B(K_{1}\sum_{j\in {𝒩}_{i}}a_{ij}({\hat{e}}_{i}(t)+e_{i}(t)-{\hat{e}}_{j}(t)-e_{j}(t))+K_{1}\sum_{o\in {𝒩}_{i}}a_{io}({\hat{e}}_{i}(t)\\ & \;\;+e_{i}(t)-e_{o}(t))+K_{1}\varphi_{i}(t)+K_{2}\sum_{k=1}^{M}c_{ik}({\hat{e}}_{i}(t)+e_{i}(t)-e_{0k}(t))+K_{3}({\hat{e}}_{i}(t)\\ & \;\;+e_{i}(t)))+K_{6}C{\hat{e}}_{i}(t)+(A+BK_{3}+BH)x_{0}^{*},\\ {\dot{\hat{e}}}_{i}(t) & \;=FCe_{i}(t)+(A+LC){\hat{e}}_{i}(t)-L\Psi_{i}(t)+FCx_{0}^{*} \end{array} \end{array}$$ (43)

by leveraging (1), (7), (8), (29–31), and (35b). For i=M+1,M+2,⋯,N, it yields that


$$\begin{array}{c} \begin{array}{cc} {\dot{e}}_{i}(t) & \;=Ae_{i}(t)+B(K_{4}\sum_{j\in {𝒩}_{i}}b_{ij}(e_{i}(t)-e_{j}(t)-{\hat{e}}_{j}(t))+K_{4}\sum_{o\in {𝒩}_{i}}b_{io}(e_{i}(t)-e_{o}(t))\\ & \;\;+K_{4}\varphi_{i}(t)+K_{5}e_{i}(t))+(A+BK_{5}+BH)x_{0}^{*}. \end{array} \end{array}$$ (44)

Let


$$\begin{array}{cc} {\hat{E}}_{0}(t) & =({\hat{e}}_{01}^{⊤}(t),{\hat{e}}_{02}^{⊤}(t),\ldots ,{\hat{e}}_{0Q}^{⊤}(t),0,0,\ldots ,0{)}^{⊤},\\ E_{0}(t) & =(e_{01}^{⊤}(t),e_{02}^{⊤}(t),\ldots ,e_{0M}^{⊤}(t){)}^{⊤},\\ E(t) & =(e_{1}^{⊤}(t),e_{2}^{⊤}(t),\ldots ,e_{N}^{⊤}(t)),\\ \hat{E}(t) & =({\hat{e}}_{1}^{⊤}(t),{\hat{e}}_{2}^{⊤}(t),\ldots ,{\hat{e}}_{M}^{⊤}(t),0,0,\ldots ,0{)}^{⊤},\\ \phi (t) & =(\phi_{1}^{⊤}(t),\phi_{2}^{⊤}(t),\ldots ,\phi_{M}^{⊤}(t){)}^{⊤},\\ \Phi (t) & =(\Phi_{1}^{⊤}(t),\Phi_{2}^{⊤}(t),\ldots ,\Phi_{M}^{⊤}(t){)}^{⊤},\\ \varphi (t) & =(\varphi_{1}^{⊤}(t),\varphi_{2}^{⊤}(t),\ldots ,\varphi_{N}^{⊤}(t)),\\ \Psi (t) & =(\Psi_{1}^{⊤}(t),\Psi_{2}^{⊤}(t),\ldots ,\Psi_{N}^{⊤}(t)). \end{array}$$

Then,


$$\begin{array}{c} \begin{array}{cc} {\dot{E}}_{0}(t) & \;=𝔸E_{0}(t)+𝔹{\hat{E}}_{0}(t)+𝔽\phi (t)+𝔾X_{0}^{*},\\ {\dot{\hat{E}}}_{0}(t) & \;=\overset{―}{F_{0}C_{0}}E_{0}(t)+(I_{\hbar }⊗(A_{0}+L_{0}C_{0})){\hat{E}}_{0}(t)-\overset{―}{L_{0}}\Phi (t)+\overset{―}{F_{0}C_{0}}X_{0}^{*},\\ \dot{E}(t) & \;=-B_{K_{2}}E_{0}(t)+\mathbb{C}E(t)+𝔻\hat{E}(t)+𝔽\phi (t)+ℍX^{*},\\ \dot{\hat{E}}(t) & \;=\overset{―}{FC}E(t)+(I_{\Theta }⊗(A+LC))\hat{E}(t)-\overset{―}{L}\Psi (t)+\overset{―}{FC}X^{*}, \end{array} \end{array}$$ (45)

where


$$\begin{array}{c} \begin{array}{c} \overset{―}{B_{0}K_{02}}=diag\{\underset{Q}{\underbrace{B_{0}K_{02},B_{0}K_{02},\ldots ,B_{0}K_{02}}},\underset{M-Q}{\underbrace{0,0,\ldots ,0}}\},\\ \overset{―}{F_{0}C_{0}}=diag\{\underset{Q}{\underbrace{F_{0}C_{0},F_{0}C_{0},\ldots ,F_{0}C_{0}}},\underset{M-Q}{\underbrace{0,0,\ldots ,0}}\},\\ \overset{―}{BK_{2}}=diag\{\underset{M}{\underbrace{BK_{2},BK_{2},\ldots ,BK_{2}}},\underset{N-M}{\underbrace{0,0,\ldots ,0}}\},\\ \overset{―}{FC}=diag\{\underset{M}{\underbrace{FC,FC,\ldots ,FC}},\underset{N-M}{\underbrace{0,0,\ldots ,0}}\},\\ \overset{―}{L_{0}}=diag\{\underset{Q}{\underbrace{L_{0},L_{0},\ldots ,L_{0}}},\underset{M-Q}{\underbrace{0,0,\ldots ,0}}\},\\ \overset{―}{L}=diag\{\underset{M}{\underbrace{L,L,\ldots ,L}},\underset{N-M}{\underbrace{0,0,\ldots ,0}}\},\\ I_{\hbar }=diag\{\underset{Q}{\underbrace{1,1,\ldots ,1}},\underset{M-Q}{\underbrace{0,0,\ldots ,0}}\},\\ I_{\Theta }=diag\{\underset{M}{\underbrace{1,1,\ldots ,1}},\underset{N-M}{\underbrace{0,0,\ldots ,0}}\},\\ 𝔸=I_{M}⊗A_{0}+(\begin{array}{c} {ℒ}_{0}⊗B_{0}K_{01}\\ {ℒ}_{0}⊗B_{0}K_{04} \end{array})+\overset{―}{B_{0}K_{02}}+(\begin{array}{cc} I_{Q}⊗B_{0}K_{03} & 0\\ 0 & I_{M-Q}⊗B_{0}K_{03} \end{array}),\\ 𝔹=(\begin{array}{cc} {ℒ}_{01}⊗B_{0}K_{01} & 0\\ {ℒ}_{02}⊗B_{0}K_{04} & 0 \end{array})+\overset{―}{B_{0}K_{02}}+\overset{―}{B_{0}K_{06}C_{0}}+(\begin{array}{cc} I_{Q}⊗B_{0}K_{03} & 0\\ 0 & 0 \end{array}),\\ B_{K_{2}}=(\begin{array}{cccc} c_{11}BK_{2} & c_{12}BK_{2} & \cdots & c_{1M}BK_{2}\\ c_{21}BK_{2} & c_{22}BK_{2} & \cdots & c_{2M}BK_{2}\\ \cdots & \cdots & \ddots & \cdots \\ c_{M1}BK_{2} & c_{M2}BK_{2} & \cdots & c_{MM}BK_{2} \end{array}), \end{array} \end{array}$$

$$\begin{array}{c} \begin{array}{c} \mathbb{C}=I_{N}⊗A+(\begin{array}{c} ℒ⊗BK_{1}\\ ℒ⊗BK_{4} \end{array})+\overset{―}{BK_{2}}+(\begin{array}{cc} I_{M}⊗BK_{3} & 0\\ 0 & I_{N-M}⊗BK_{3} \end{array}),\\ 𝔼=(\begin{array}{cc} I_{Q}⊗B_{0}K_{01} & 0\\ 0 & I_{M-Q}⊗B_{0}K_{04} \end{array}),\\ 𝔻=(\begin{array}{cc} {ℒ}_{1}⊗BK_{1} & 0\\ {ℒ}_{2}⊗BK_{4} & 0 \end{array})+\overset{―}{BK_{2}}+\overset{―}{BK_{6}C}+(\begin{array}{cc} I_{M}⊗BK_{3} & 0\\ 0 & 0 \end{array}),\\ 𝔽=(\begin{array}{cc} I_{M}⊗BK_{1} & 0\\ 0 & I_{N-M}⊗BK_{4} \end{array}),\\ 𝔾=I_{M}⊗A_{0}+(\begin{array}{cc} I_{Q}⊗B_{0}K_{03}+B_{0}G & 0\\ 0 & I_{M-Q}⊗B_{0}K_{05}+B_{0}G \end{array}),\\ ℍ=I_{N}⊗A+(\begin{array}{cc} I_{M}⊗BK_{3}+BH & 0\\ 0 & I_{N-M}⊗BK_{5}+BH \end{array}). \end{array} \end{array}$$

Define $\tilde{E}(t)=(E_{0}^{⊤}(t),{\hat{E}}_{0}^{⊤}(t),E^{⊤}(t),{\hat{E}}^{⊤}(t){)}^{⊤}$. Then,


$$\begin{array}{cc} \dot{\tilde{E}}(t) & =(\begin{array}{cccc} 𝔸 & 𝔹 & 0 & 0\\ \overset{―}{F_{0}C_{0}} & I_{\hbar }⊗(A_{0}+L_{0}C_{0}) & 0 & 0\\ -B_{K_{2}} & 0 & \mathbb{C} & 𝔻\\ 0 & 0 & \overset{―}{FC} & I_{\Theta }⊗(A+LC) \end{array})\\ & \;\times \tilde{E}(t)+(\begin{array}{cccc} 𝔼 & 0 & 0 & 0\\ 0 & -{\overset{―}{L}}_{0} & 0 & 0\\ 0 & 0 & 𝔽 & 0\\ 0 & 0 & 0 & -\overset{―}{L} \end{array})(\begin{array}{c} \phi (t)\\ \Phi (t)\\ \varphi (t)\\ \Psi (t) \end{array})(\begin{array}{cccc} 𝔾 & 0 & 0 & 0\\ \overset{―}{F_{0}C_{0}} & 0 & 0 & 0\\ 0 & 0 & ℍ & 0\\ 0 & 0 & \overset{―}{FC} & 0 \end{array})(\begin{array}{c} X_{0}^{*}\\ 0\\ X^{*}\\ 0 \end{array}). \end{array}$$ (46)

With initial conditions $E_{0}(t_{0})⪰0$, ${\hat{E}}_{0}(t_{0})⪰0$, $E(t_{0})⪰0$, and $\hat{E}(t_{0})⪰0$, it follows from (11d), (38), and (45) that


$$\begin{array}{c} \begin{array}{cc} {\dot{E}}_{0}(t_{0}) & \;⪰𝕀E_{0}(t_{0})+𝔹{\hat{E}}_{0}(t_{0})+𝔾X_{0}^{*},\\ {\dot{\hat{E}}}_{0}(t_{0}) & \;⪰(\overset{―}{F_{0}C_{0}}+\overset{―}{\pi }\overset{―}{L_{0}1_{q\times q}C_{0}})E_{0}(t_{0})+(I_{\hbar }⊗(A_{0}\\ & \;\;+L_{0}C_{0})){\hat{E}}_{0}(t_{0})+(\overset{―}{F_{0}C_{0}}+\overset{―}{\pi }\overset{―}{L_{0}1_{q\times q}C_{0}})X_{0}^{*},\\ \dot{E}(t_{0}) & \;⪰-B_{K_{2}}E_{0}(t_{0})+𝕁E(t_{0})+𝔻\hat{E}(t_{0})+ℍX^{*},\\ \dot{\hat{E}}(t_{0}) & \;⪰(\overset{―}{FC}+\overset{―}{\omega }\overset{―}{L1_{q\times q}C})E(t_{0})+(I_{\Theta }⊗(A+LC))\\ & \;\;\times \hat{E}(t_{0})+(\overset{―}{FC}+\overset{―}{\omega }\overset{―}{L1_{q\times q}C})X^{*}, \end{array} \end{array}$$ (47)

where


$$\begin{array}{c} \begin{array}{c} \overset{―}{L_{0}1_{q\times q}C_{0}}=diag\{\underset{Q}{\underbrace{L_{0}1_{q\times q}C_{0},\ldots ,L_{0}1_{q\times q}C_{0}}},\underset{M-Q}{\underbrace{0,0,\ldots ,0}}\},\\ \overset{―}{L1_{q\times q}C}=diag\{\underset{M}{\underbrace{L1_{q\times q}C,\ldots ,L1_{q\times q}C}},\underset{N-M}{\underbrace{0,0,\ldots ,0}}\},\\ 𝕀=I_{M}⊗A_{0}+(\begin{array}{c} {ℒ}_{0}⊗B_{0}K_{01}\\ {ℒ}_{0}⊗B_{0}K_{04} \end{array})+\overset{―}{B_{0}K_{02}}+(\begin{array}{cc} {\bar{B}}_{Q0} & 0\\ 0 & {\bar{B}}_{MQ0} \end{array}),\\ 𝕁=I_{N}⊗A+(\begin{array}{c} ℒ⊗BK_{1}\\ ℒ⊗BK_{4} \end{array})+\overset{―}{BK_{2}}+(\begin{array}{cc} {\bar{B}}_{M} & 0\\ 0 & {\bar{B}}_{NM} \end{array}),\\ {\bar{B}}_{Q0}=I_{Q}⊗B_{0}K_{03}+\overset{―}{\alpha }I_{Q}⊗B_{0}K_{01}1_{n\times n},\\ {\bar{B}}_{MQ0}=I_{M-Q}⊗B_{0}K_{05}+\overset{―}{\alpha }I_{M-Q}⊗B_{0}K_{04}1_{n\times n},\\ {\bar{B}}_{M}=I_{M}⊗BK_{3}+\overset{―}{\nu }I_{M}⊗BK_{1}1_{n\times n},\\ {\bar{B}}_{NM}=I_{N-M}⊗BK_{5}+\overset{―}{\nu }I_{N-M}⊗BK_{4}1_{n\times n}. \end{array} \end{array}$$

According to (46) and (47), one can derive


$$\begin{array}{c} \begin{array}{cc} \dot{\tilde{E}}(t_{0}) & \;⪰(\begin{array}{cccc} 𝕀 & 𝔹 & 0 & 0\\ {ℱ}_{0} & {𝒜}_{0} & 0 & 0\\ -B_{K_{2}} & 0 & 𝕁 & 𝔻\\ 0 & 0 & ℱ & I_{\Theta }⊗(A+LC) \end{array})\tilde{E}(t_{0})+(\begin{array}{cccc} 𝔾 & 0 & 0 & 0\\ {ℱ}_{0} & 0 & 0 & 0\\ 0 & 0 & ℍ & 0\\ 0 & 0 & ℱ & 0 \end{array})(\begin{array}{c} X_{0}^{*}\\ 0\\ X^{*}\\ 0 \end{array}), \end{array} \end{array}$$ (48)

where ${ℱ}_{0}=\overset{―}{F_{0}C_{0}}+\overset{―}{\pi }\overset{―}{L_{0}1_{q\times q}C_{0}}$, ${𝒜}_{0}=I_{\hbar }⊗(A_{0}+L_{0}C_{0})$, $ℱ=\overset{―}{FC}+\overset{―}{\omega }\overset{―}{L1_{q\times q}C}$.

Together with (35a), (35b), (36), and Lemma 3 gives $A_{0}+\sum_{s\in {ℳ}_{k}}b_{ks}B_{0}K_{01}+B_{0}K_{02}+B_{0}K_{03}+\overset{―}{\alpha }B_{0}K_{01}1_{n\times n}$ and $A_{0}+\sum_{l\in {ℳ}_{k}}b_{kl}B_{0}K_{04}+B_{0}K_{05}+\overset{―}{\alpha }B_{0}K_{04}1_{n\times n}$ are Metzler. Hence, 𝕀 is Metzler. Through (35c), (35d) and (36), it can be given that $A_{0}+L_{0}C_{0}$ and *A* + *LC* are also Metzler by virtue of Lemma 3. Furthermore, $I_{\hbar }⊗(A_{0}+L_{0}C_{0})$ and $I_{\Theta }⊗(A+LC)$ are Metzler.

It follows from (35e), (35f), (36), Lemma 3 that $A+\sum_{j\in {𝒩}_{i}}a_{ij}BK_{1}+BK_{2}+BK_{3}+\overset{―}{\nu }BK_{1}1_{n\times n}$ and $A+\sum_{o\in {𝒩}_{i}}a_{io}BK_{4}+BK_{5}+\overset{―}{\nu }BK_{4}1_{n\times n}$ are Metzler. It means that 𝕁 is Metzler.

By (35g), (35h), and (36), one has $\sum_{s\in {ℳ}_{1}}b_{ks}B_{0}K_{01}+B_{0}K_{02}+B_{0}K_{06}+B_{0}K_{03}⪰0$ and $\sum_{j\in {𝒩}_{i}}a_{ij}BK_{1}+BK_{2}+BK_{6}+BK_{3}⪰0$. Then, we can get 𝔹⪰0 and 𝔻⪰0. Together with (35i), (35j), and (36) yields $F_{0}C_{0}+\overset{―}{\pi }L_{0}1_{q\times q}C_{0}⪰0$ and $FC+\overset{―}{\omega }L1_{q\times q}C⪰0$. Furthermore, ${ℱ}_{0}⪰0$ and ℱ⪰0. Using (35k), (35l), and (36) gives $(A_{0}+B_{0}K_{03}+B_{0}G)x_{0}^{*}⪰0$ and $(A_{0}+B_{0}K_{05}+B_{0}G)x_{0}^{*}⪰0$. Through (35m), (35n), and (36), it is known that $(A+BK_{3}+BH)x_{0}^{*}⪰0$ and $(A+BK_{3}+BH)x_{0}^{*}⪰0$. Consequently, 𝔾⪰0 and ℍ⪰0. Since ζ≺0, then $B_{K_{2}}≺0$ holds, that is, $-B_{K_{2}}≻0$. By Lemma 1, one has $\dot{\tilde{E}}(t_{0})⪰0$ for $\tilde{E}(t)⪰0$, so $\tilde{E}(t)⪰0$. Moreover, for any initial condition $\tilde{E}(t_{0})⪰0$, it is obtained that $\tilde{E}(t)⪰0$. Therefore, the system (46) is positive.

Next, the consensus of system (46) is considered. From (11d), (38), and (45), the following inequalities are deduced.


$$\begin{array}{c} \begin{array}{cc} {\dot{E}}_{0}(t) & \;⪯𝕂E_{0}(t)+𝔹{\hat{E}}_{0}(t)+𝔾X_{0}^{*},\\ {\dot{\hat{E}}}_{0}(t) & \;⪯(\overset{―}{F_{0}C_{0}}-\overset{―}{\pi }\overset{―}{L_{0}1_{q\times q}C_{0}})E_{0}(t)+(I_{\hbar }⊗(A_{0}\\ & \;\;+L_{0}C_{0})){\hat{E}}_{0}(t)+(\overset{―}{F_{0}C_{0}}-\overset{―}{\pi }\overset{―}{L_{0}1_{q\times q}C_{0}})X_{0}^{*},\\ \dot{E}(t) & \;⪯-B_{K_{2}}E_{0}(t)+𝕃E(t)+𝔻\hat{E}(t)+ℍX^{*},\\ \dot{\hat{E}}(t) & \;⪯(\overset{―}{FC}-\overset{―}{\omega }\overset{―}{L1_{q\times q}C})E(t)+(I_{\Theta }⊗(A+LC))\\ & \;\;\times \hat{E}(t)+(\overset{―}{FC}-\overset{―}{\omega }\overset{―}{L1_{q\times q}C})X^{*}, \end{array} \end{array}$$ (49)

where


$$\begin{array}{c} \begin{array}{c} 𝕂=I_{M}⊗A_{0}+(\begin{array}{c} {ℒ}_{0}⊗B_{0}K_{01}\\ {ℒ}_{0}⊗B_{0}K_{04} \end{array})+\overset{―}{B_{0}K_{02}}+(\begin{array}{cc} \begin{array}{c} {\tilde{B}}_{Q0} \end{array} & 0\\ 0 & {\tilde{B}}_{MQ0} \end{array}),\\ 𝕃=I_{N}⊗A+(\begin{array}{c} ℒ⊗BK_{1}\\ ℒ⊗BK_{4} \end{array})+\overset{―}{BK_{2}}+(\begin{array}{cc} {\tilde{B}}_{M} & 0\\ 0 & {\tilde{B}}_{NM} \end{array}),\\ {\tilde{B}}_{Q0}=I_{Q}⊗B_{0}K_{03}-\overset{―}{\alpha }I_{Q}⊗B_{0}K_{01}1_{n\times n},\\ {\tilde{B}}_{MQ0}=I_{M-Q}⊗B_{0}K_{05}-\overset{―}{\alpha }I_{M-Q}⊗B_{0}K_{04}1_{n\times n},\\ {\tilde{B}}_{M}=I_{M}⊗BK_{3}-\overset{―}{\nu }I_{M}⊗BK_{1}1_{n\times n},\\ {\tilde{B}}_{NM}=I_{N-M}⊗BK_{5}-\overset{―}{\nu }I_{N-M}⊗BK_{4}1_{n\times n}. \end{array} \end{array}$$

In the light of (49), it follows that


$$\begin{array}{c} \begin{array}{cc} \dot{\tilde{E}}(t_{0}) & \;⪯(\begin{array}{cccc} 𝕂 & 𝔹 & 0 & 0\\ {\bar{ℱ}}_{0} & {𝒜}_{0} & 0 & 0\\ -B_{K_{2}} & 0 & 𝕃 & 𝔻\\ 0 & 0 & \bar{ℱ} & I_{\Theta }⊗(A+LC) \end{array})\tilde{E}(t_{0})+(\begin{array}{cccc} 𝔾 & 0 & 0 & 0\\ {\bar{ℱ}}_{0} & 0 & 0 & 0\\ 0 & 0 & ℍ & 0\\ 0 & 0 & \bar{ℱ} & 0 \end{array})(\begin{array}{c} X_{0}^{*}\\ 0\\ X^{*}\\ 0 \end{array}) \end{array} \end{array}$$ (50)

with ${\bar{ℱ}}_{0}=\overset{―}{F_{0}C_{0}}-\overset{―}{\pi }\overset{―}{L_{0}1_{q\times q}C_{0}}$ and $\bar{ℱ}=\overset{―}{FC}-\overset{―}{\omega }\overset{―}{L1_{q\times q}C}$.

Choose a CLF $V(\tilde{E}(t))={\tilde{E}}^{⊤}(t)\vartheta =E_{0}^{⊤}(t)(1_{M}⊗v_{0})+{\hat{E}}_{0}^{⊤}(t)(1_{M}⊗v_{1})+E^{⊤}(t)(1_{N}⊗v_{2})+{\hat{E}}^{⊤}(t)(1_{N}⊗v_{3})$. By (50), it yields that


$$\begin{array}{cc} \dot{V}(\tilde{E}(t)) & \leq E_{0}^{⊤}(t)({𝕂}^{⊤}(1_{M}⊗v_{0})+{\bar{ℱ}}_{0}^{⊤}(1_{M}⊗v_{1})-B_{K_{2}}^{⊤}(1_{N}⊗v_{2}))\\ & \;+{\hat{E}}_{0}^{⊤}(t)({𝔹}^{⊤}(1_{M}⊗v_{0})+{𝒜}_{0}^{⊤}(1_{M}⊗v_{1}))+E^{⊤}(t)({𝕃}^{⊤}(1_{N}⊗v_{2})\\ & \;+{\bar{ℱ}}^{⊤}(1_{N}⊗v_{3}))+{\hat{E}}^{⊤}(t)({𝔻}^{⊤}(1_{N}⊗v_{2})+I_{\Theta }⊗(A+LC{)}^{⊤} \end{array}$$ (51)

$$\begin{array}{cc} & \;\times (1_{N}⊗v_{3}))+(X_{0}^{*}{)}^{⊤}({𝔾}^{⊤}(1_{M}⊗v_{0})+{\bar{ℱ}}_{0}^{⊤}(1_{M}⊗v_{1}))\\ & \;+(X^{*}{)}^{⊤}({ℍ}^{⊤}(1_{N}⊗v_{2})+{\bar{ℱ}}^{⊤}(1_{N}⊗v_{3})), \end{array}$$

where


$$\begin{array}{c} \begin{array}{cc} & \;\;{𝕂}^{⊤}(1_{M}⊗v_{0})+{\bar{ℱ}}_{0}^{⊤}(1_{M}⊗v_{1})-B_{K_{2}}^{⊤}(1_{N}⊗v_{2})\\ & \;=[\Theta_{11}^{⊤},\Theta_{12}^{⊤},\cdots ,\Theta_{1Q}^{⊤},\Theta_{2(Q+1)},\cdots ,\Theta_{2M}^{⊤}{]}^{⊤},\\ \Theta_{1k} & \;=A_{0}^{⊤}v_{0}+\sum_{s\in {ℳ}_{k}}a_{1k}K_{01}^{⊤}B_{0}^{⊤}v_{0}-\sum_{s\in {ℳ}_{k}}a_{sk}K_{01}^{⊤}B_{0}^{⊤}v_{0}-\sum_{l\in {ℳ}_{k}}b_{lk}K_{04}^{⊤}B_{0}^{⊤}v_{0}\\ & \;\;+K_{02}^{⊤}B_{0}^{⊤}v_{0}+K_{03}^{⊤}B_{0}^{⊤}v_{0}-\overset{―}{\alpha }1_{n\times n}K_{01}^{⊤}B_{0}^{⊤}v_{0}+C_{0}^{⊤}F_{0}^{⊤}v_{1}-\overset{―}{\pi }C_{0}^{⊤}1_{q\times q}L_{0}^{⊤}v_{1}\\ & \;\;-K_{2}^{⊤}B^{⊤}v_{2},\;k=1,\cdots ,Q,\\ \Theta_{2k} & \;=A_{0}^{⊤}v_{0}+\sum_{l\in {ℳ}_{k}}b_{kl}K_{04}^{⊤}B_{0}^{⊤}v_{0}-\sum_{l\in {ℳ}_{k}}b_{lk}K_{04}^{⊤}B_{0}^{⊤}v_{0}-\sum_{s\in {ℳ}_{k}}a_{sk}K_{01}^{⊤}B_{0}^{⊤}v_{0}\\ & \;\;+K_{05}^{⊤}B_{0}^{⊤}v_{0}-\overset{―}{\alpha }1_{n\times n}K_{04}^{⊤}B_{0}^{⊤}v_{0},\;k=Q+1,\cdots ,M,\\ & \;\;{𝔹}^{⊤}(1_{M}⊗v_{0})+{𝒜}_{0}^{⊤}(1_{M}⊗v_{1})=[\Theta_{31}^{⊤},\Theta_{32}^{⊤},\cdots ,\Theta_{3Q}^{⊤},0,\cdots ,0{]}^{⊤},\\ \Theta_{3k} & \;=\sum_{s\in {ℳ}_{k}}a_{ks}K_{01}^{⊤}B_{0}^{⊤}v_{0}-\sum_{s\in {ℳ}_{k}}a_{sk}K_{01}^{⊤}B_{0}^{⊤}v_{0}-\sum_{l\in {ℳ}_{k}}b_{lk}K_{04}^{⊤}B_{0}^{⊤}v_{0}\\ & \;\;+K_{02}^{⊤}B_{0}^{⊤}v_{0}+K_{06}^{⊤}B_{0}^{⊤}v_{0}+K_{03}^{⊤}B_{0}^{⊤}v_{0}+A_{0}^{⊤}v_{1}+C_{0}^{⊤}L_{0}^{⊤}v_{1},\;k=1,2,\cdots ,Q,\\ & \;\;{𝕃}^{⊤}(1_{N}⊗v_{2})+{\bar{ℱ}}^{⊤}(1_{N}⊗v_{3})=[\Gamma_{11}^{⊤},\Gamma_{12}^{⊤},\cdots ,\Gamma_{1M}^{⊤},\Gamma_{2(M+1)}^{⊤},\cdots ,\Gamma_{2N}^{⊤}{]}^{⊤},\\ \Gamma_{1i} & \;=A^{⊤}v_{2}+\sum_{j\in {𝒩}_{i}}a_{ij}K_{1}^{⊤}B^{⊤}v_{2}-\sum_{j\in {𝒩}_{i}}a_{ji}K_{1}^{⊤}B^{⊤}v_{2}-\sum_{o\in {𝒩}_{i}}b_{oi}K_{4}^{⊤}B^{⊤}v_{2}\\ & \;\;+K_{2}^{⊤}B^{⊤}v_{2}+K_{3}^{⊤}B^{⊤}v_{2}-\overset{―}{\nu }1_{n\times n}K_{1}^{⊤}B^{⊤}v_{2}+C^{⊤}F^{⊤}v_{3}\\ & \;\;-\overset{―}{\omega }C^{⊤}1_{q\times q}L^{⊤}v_{3},\;i=1,2,\cdots ,M,\\ \Gamma_{2i} & \;=A^{⊤}v_{2}+\sum_{o\in {𝒩}_{i}}b_{io}K_{4}^{⊤}B^{⊤}v_{2}-\sum_{o\in {𝒩}_{i}}b_{oi}K_{4}^{⊤}B^{⊤}v_{2}-\sum_{j\in {𝒩}_{i}}a_{ji}K_{1}^{⊤}B^{⊤}v_{2}\\ & \;\;+K_{5}^{⊤}B^{⊤}v_{2}-\overset{―}{\nu }1_{n\times n}K_{4}^{⊤}B^{⊤}v_{2},\;i=M+1,\cdots ,N,\\ & \;\;{𝔻}^{⊤}(1_{N}⊗v_{2})+I_{\Theta }⊗(A+LC{)}^{⊤}(1_{N}⊗v_{3})=[\Gamma_{31}^{⊤},\Gamma_{32}^{⊤},\cdots ,\Gamma_{3M}^{⊤},0,\cdots ,0{]}^{⊤},\\ \Gamma_{3i} & \;=\sum_{j\in {𝒩}_{i}}a_{ij}K_{1}^{⊤}B^{⊤}v_{2}-\sum_{j\in {𝒩}_{i}}a_{ji}K_{1}^{⊤}B^{⊤}v_{2}-\sum_{o\in {𝒩}_{i}}b_{oi}K_{4}^{⊤}B^{⊤}v_{2}\\ & \;\;+K_{2}^{⊤}B^{⊤}v_{2}+K_{6}^{⊤}B^{⊤}v_{2}+K_{3}^{⊤}B^{⊤}v_{2}+A^{⊤}v_{3}+C^{⊤}L^{⊤}v_{3},\;i=1,2,\cdots ,M,\\ & \;\;(X_{0}^{*}{)}^{⊤}({𝔾}^{⊤}(1_{M}⊗v_{0})+{\bar{ℱ}}_{0}^{⊤}(1_{M}⊗v_{1}))+(X^{*}{)}^{⊤}({ℍ}^{⊤}(1_{N}⊗v_{2})+{\bar{ℱ}}^{⊤}(1_{N}⊗v_{3}))\\ & \;=(X^{*}{)}^{⊤}(ℜ⊗A_{0}^{⊤}v_{0}+ℵ⊗K_{03}^{⊤}B_{0}^{⊤}v_{0}+℧⊗K_{05}^{⊤}B_{0}^{⊤}v_{0}+ℜ⊗G^{⊤}B_{0}^{⊤}v_{0}\\ & \;\;+ℵ⊗C_{0}^{⊤}F_{0}^{⊤}v_{1}-\overset{―}{\pi }ℵ⊗C_{0}^{⊤}1_{q\times q}L_{0}^{⊤}v_{1}+1_{N}⊗A^{⊤}v_{2}+ℜ⊗K_{3}^{⊤}B^{⊤}v_{2}\\ & \;\;+ℷ⊗K_{5}^{⊤}B^{⊤}v_{2}+1_{N}⊗H^{⊤}B^{⊤}v_{2}+ℜ⊗C^{⊤}F^{⊤}v_{3}-\overset{―}{\omega }ℜ⊗C^{⊤}1_{q\times q}L^{⊤}v_{3}). \end{array} \end{array}$$

By (35v) and (36), it derives that the following inequalities


$$\begin{array}{c} K_{01}⪯\frac{1_{r}\beta^{⊤}}{1_{r}^{⊤}B_{0}^{⊤}v_{0}},K_{02}⪯\frac{1_{r}\gamma^{⊤}}{1_{r}^{⊤}B_{0}^{⊤}v_{0}},K_{03}⪯\frac{1_{r}\eta^{⊤}}{1_{r}^{⊤}B_{0}^{⊤}v_{0}},K_{04}⪯\frac{1_{r}\kappa^{⊤}}{1_{r}^{⊤}B_{0}^{⊤}v_{0}},K_{05}⪯\frac{1_{r}\xi^{⊤}}{1_{r}^{⊤}B_{0}^{⊤}v_{0}},\\ K_{06}⪯\frac{1_{r}\psi^{⊤}}{1_{r}^{⊤}B_{0}^{⊤}v_{0}},G⪯\frac{1_{r}\pi^{⊤}}{1_{r}^{⊤}B_{0}^{⊤}v_{0}},K_{1}⪯\frac{1_{r}\delta^{⊤}}{1_{r}^{⊤}B^{⊤}v_{2}},K_{2}⪯\frac{1_{r}\zeta^{⊤}}{1_{r}^{⊤}B^{⊤}v_{2}},K_{3}⪯\frac{1_{r}\theta^{⊤}}{1_{r}^{⊤}B^{⊤}v_{2}},\\ K_{4}⪯\frac{1_{r}o^{⊤}}{1_{r}^{⊤}B^{⊤}v_{2}},K_{5}⪯\frac{1_{r}\varrho^{⊤}}{1_{r}^{⊤}B^{⊤}v_{2}},K_{6}⪯\frac{1_{r}\chi^{⊤}}{1_{r}^{⊤}B^{⊤}v_{2}},H⪯\frac{1_{r}\rho^{⊤}}{1_{r}^{⊤}B^{⊤}v_{2}},F_{0}⪯\frac{1_{n}\sigma_{ı}^{⊤}}{1_{n}^{⊤}v_{1}},\\ L_{0}⪯\frac{1_{n}\varsigma_{ı}^{⊤}}{1_{n}^{⊤}v_{1}},F⪯\frac{1_{n}\tau_{ı}^{⊤}}{1_{n}^{⊤}v_{3}},L⪯\frac{1_{n}\varepsilon_{ı}^{⊤}}{1_{n}^{⊤}v_{3}}. \end{array}$$

Then, we have


$$\begin{array}{c} \begin{array}{cc} \Theta_{1k} & \;⪯A_{0}^{⊤}v_{0}+\sum_{s\in {ℳ}_{k}}a_{ks}\beta -\sum_{s\in {ℳ}_{k}}a_{sk}\beta -\sum_{l\in {ℳ}_{k}}b_{lk}\\ & \;\;\times \kappa +\gamma +\eta -\overset{―}{\alpha }1_{n\times n}\beta +C_{0}^{⊤}\sigma -\overset{―}{\pi }C_{0}^{⊤}1_{q\times q}\varsigma -\zeta ,\\ \Theta_{2k} & \;⪯A_{0}^{⊤}v_{0}+\sum_{l\in {ℳ}_{k}}b_{kl}\kappa -\sum_{l\in {ℳ}_{k}}b_{lk}\kappa \\ & \;\;-\sum_{s\in {ℳ}_{k}}a_{sk}\beta +\xi -\overset{―}{\alpha }1_{n\times n}\kappa ,\\ \Theta_{3k} & \;⪯\sum_{s\in {ℳ}_{k}}a_{ks}\beta -\sum_{s\in {ℳ}_{k}}a_{sk}\beta -\sum_{l\in {ℳ}_{k}}b_{lk}\kappa \\ & \;\;+\gamma +\psi +\eta +A_{0}^{⊤}v_{1}+C_{0}^{⊤}\varsigma ,\\ \Gamma_{1i} & \;⪯A^{⊤}v_{2}+\sum_{j\in {𝒩}_{i}}a_{ij}\delta -\sum_{j\in {𝒩}_{i}}a_{ji}\delta -\sum_{o\in {𝒩}_{i}}b_{oi}o\\ & \;\;+\zeta +\theta -\overset{―}{\nu }1_{n\times n}\delta +C^{⊤}\tau -\overset{―}{\omega }C^{⊤}1_{q\times q}\varepsilon ,\\ \Gamma_{2i} & \;⪯A^{⊤}v_{2}+\sum_{o\in {𝒩}_{i}}a_{io}o-\sum_{o\in {𝒩}_{i}}a_{oi}o\\ & \;\;-\sum_{j\in {𝒩}_{i}}a_{ji}\delta +\varrho -\overset{―}{\nu }1_{n\times n}o,\\ \Gamma_{3i} & \;⪯\sum_{j\in {𝒩}_{i}}a_{ij}\delta -\sum_{j\in {𝒩}_{i}}a_{ji}\delta -\sum_{o\in {𝒩}_{i}}b_{oi}o+\zeta +\chi \\ & \;\;+\theta +A^{⊤}v_{3}+C^{⊤}\varepsilon , \end{array} \end{array}$$

$$\begin{array}{c} \begin{array}{cc} & \;\;(X_{0}^{*}{)}^{⊤}({𝔾}^{⊤}(1_{M}⊗v_{0})+{\bar{ℱ}}_{0}^{⊤}(1_{M}⊗v_{1}))\\ & \;\;+(X^{*}{)}^{⊤}({ℍ}^{⊤}(1_{N}⊗v_{2})+{\bar{ℱ}}^{⊤}(1_{N}⊗v_{3}))\\ & \;\leq (X^{*}{)}^{⊤}(ℜ⊗A_{0}^{⊤}v_{0}+ℵ⊗\eta +℧⊗\xi +ℜ⊗\pi +ℵ⊗\\ & \;\;C_{0}^{⊤}\sigma -\overset{―}{\pi }ℵ⊗C_{0}^{⊤}1_{q\times q}\varsigma +1_{N}⊗A^{⊤}v_{2}+ℜ⊗\theta \\ & \;\;+ℷ⊗\varrho +1_{N}⊗\rho +ℜ⊗C^{⊤}\tau -\overset{―}{\omega }ℜ⊗C^{⊤}1_{q\times q}\varepsilon ). \end{array} \end{array}$$

Furthermore, the condition (51) can be rewritten as $\dot{V}(t)<-\varpi_{1}{\tilde{E}}^{⊤}(t)\vartheta +\varpi_{2}(X^{*}{)}^{⊤}(1_{N}⊗v_{a})$ by (35o-35u). This refers to the fact that $\lim_{t\to \infty }\|x_{0}(t)-x_{0}^{*}{\|}_{1}<\Omega$, where $\Omega =\frac{\varpi_{2}}{\hbar \varpi_{1}}(X^{*}{)}^{⊤}(1_{N}⊗v_{a})$, $\hbar =\min_{\ell \in \{1,2,\cdots ,n\}}\{v_{0\ell }\}$, and $v_{0\ell }$ is the component of *v*_0_. Therefore, based on Definition 2, the practical consensus of system (46) is guaranteed. ∎

**Remark 7** The literature [19–24] have explored various consensus problems in multi-agent systems utilizing the pinning control strategy. It is noteworthy that the pinning control strategies adopted in the aforementioned literature typically presuppose the availability of system states. However, in practical applications, system states may not be readily accessible due to various external factors. Therefore, it is crucial to observe the system states promptly and accurately. In light of this consideration, Theorem 2 proposes an observer based on the pinning control strategy, which monitors only the pinned agents and not the unpinned ones. This approach reduces the resource consumption during the observation process.

**Remark 8** The literature [32–34] investigated double event-triggered control for MASs. The proposed ETM effectively reduced the frequency of communication among agents and the update of controllers. However, the event-triggered observers were not taken into account. Consequently, this paper constructs a double ETM for both observers and controllers. Adaptive event-triggered observers and controllers are designed for the followers in PMASs, respectively. Additionally, a double adaptive ETM for the leader is also considered, which further diminishes the consumption of communication resources. Compared with the quadratic method-based ETM in [32–34], the ETM proposed in this paper is more suitable for PMASs.

## 4 Discussion on quantization effects

Common quantization effects fall into two main categories: quantization communication [38] and input quantization [39]. In order to deal with the unavoidable constraints in networks, quantization control methods are used as an effective control strategy to handle these constraints and reduce communication overhead. In recent years, researchers have generally used logarithmic quantizers [38] or uniform quantizers to solve communication quantization problems, while input quantization problems are handled by using hysteresis quantizers [39].

Since quantization control is a complex and extensive research topic in the field of control, this section will briefly discuss communication quantization problems using logarithmic quantizers as an example. A definition of logarithmic quantizers and possible quantization controller designs are given. More detailed consistency analysis and control gain design will be discussed in detail in our future work.

A logarithmic quantizer $q_{l}:\mathbb{R}\to {\tilde{\varpi }}_{i}$ can be described as


$$\begin{array}{c} q_{l}(x)=\left\{ \begin{array}{cc} \varpi_{i}, & if\;\frac{1}{1+\vartheta }\varpi_{i}<x\leq \frac{1}{1-\vartheta }\varpi_{i},\\ 0, & if\;x=0,\\ -q_{l}(-x), & if\;x<0, \end{array}\right. \end{array}$$ (52)

where $\varpi_{i}$ is the quantization level and the quantization parameter ϑ satisfies ϑ∈(0,1/3). Denoted the set of quantized level by


$${\tilde{\varpi }}_{i}=\{\pm \varpi_{i}:\varpi_{i}=(\frac{1-\vartheta }{1+\vartheta }{)}^{i}\varpi_{0},i=\pm 1,\pm 2,\cdots ,\}\cup \pm \varpi_{0}\cup 0,\;\varpi_{0}>0.$$

Based on the concept of quantizers, it can be obtained that $\left|q_{l}(r)-r|\leq \vartheta |r\right|$, ∀r∈ℝ. Define $\bar{ℸ}=[{\bar{ℸ}}_{1},{\bar{ℸ}}_{2},\cdots ,{\bar{ℸ}}_{n}{]}^{⊤}\in {\mathbb{R}}^{n}$ and $q_{l}(\bar{ℸ})=[q_{l}({\bar{ℸ}}_{1}),q_{l}({\bar{ℸ}}_{2}),\cdots ,q_{l}({\bar{ℸ}}_{n}){]}^{⊤}$, one has $q_{l}(\bar{ℸ})-\bar{ℸ}=ℋ\bar{ℸ}$ with $ℋ=diag\{{ℋ}_{1},{ℋ}_{2},\cdots ,{ℋ}_{n}\}$ and ${ℋ}_{i}\in [-\vartheta ,\vartheta ]$.

Combining multi-adaptive event-triggered mechanisms in section 0.2, the quantization controllers for leaders and followers can be designed as follows:


$$\begin{array}{c} u_{0k}(t)={\bar{K}}_{01}z_{0k}(t_{p}^{k})+{\bar{K}}_{02}q_{l}((x_{0k}(t)-x_{0}^{*}))\\ \;+{\bar{K}}_{03}x_{0k}(t)+\bar{G}x_{0}^{*},\;k=1,2,\ldots ,Q,\\ u_{0k}(t)={\bar{K}}_{01}z_{0k}(t_{p}^{k})+{\bar{K}}_{03}x_{0k}(t)+\bar{G}x_{0}^{*},k=Q+1,Q+2,\ldots ,M \end{array}$$ (53)

and


$$\begin{array}{c} u_{i}(t)={\bar{K}}_{1}z_{i}(t_{\sigma }^{i})+{\bar{K}}_{2}q_{l}(\sum_{k=1}^{M}c_{ik}(x_{i}(t)-x_{0k}(t)))\\ \;+{\bar{K}}_{3}x_{i}(t)+\bar{H}x_{0}^{*},\;i=1,2,\ldots ,M,\;\\ u_{i}(t)={\bar{K}}_{1}z_{i}(t_{\sigma }^{i})+{\bar{K}}_{3}x_{i}(t)+\bar{H}x_{0}^{*},i=M+1,M+2,\ldots ,N, \end{array}$$ (54)

where $z_{0k}(t_{p}^{k})$ and $z_{i}(t_{\sigma }^{i})$ are defined as


$$\begin{array}{c} z_{0k}(t_{p}^{k})=q_{l}(\sum_{s\in {ℳ}_{k}}b_{ks}(x_{0k}(t_{p}^{k})-x_{0s}(t_{p}^{k}))),\\ z_{i}(t_{\sigma }^{i})=q_{l}(\sum_{j\in {𝒩}_{i}}a_{ij}(x_{i}(t_{\sigma }^{i})-x_{j}(t_{\sigma }^{i}))). \end{array}$$ (55)

Correspondingly, the design of multi-adaptive event-triggered mechanisms should also be changed to a version based on quantization information.

## 5 Illustrative examples

**Fig 1:**
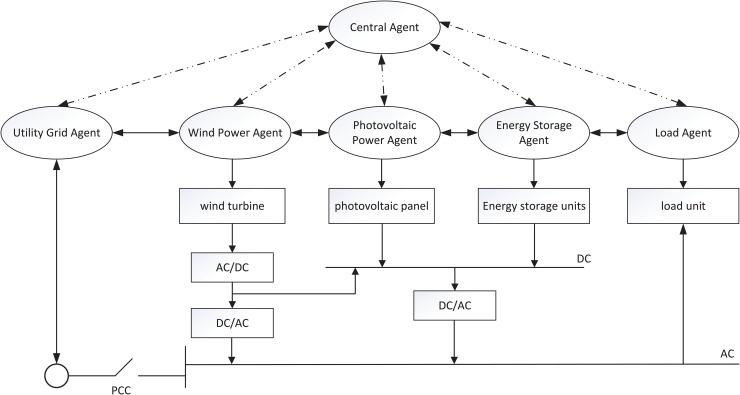
Structure of MASs-based microgrids.

Fossil energy sources are becoming increasingly depleted globally and the pollution problems associated with energy consumption are becoming more and more serious. At the same time, the development of microgrids based on renewable energy sources such as solar and wind power has begun to grow rapidly. The traditional control method is centralized control, which mainly relies on a central controller. With the increasing number of micro-sources, failure of the central controller will lead to failure of control or even paralysis of the entire power system. It is considered that the microgrid consists of multiple components, where different components have different control instructions and need to cooperate to achieve power generation. Meanwhile, one component is needed to issue control commands based on information from all components, so that coordinated control is accomplished. There are many similarities between MASs and microgrids. Therefore, it can be modeled as MASs. The literature [40] designed an intermittent dynamic time interval pinning control strategy for application to distributed control of microgrids, where each component in the microgrid is considered as an agent. Fig 1 shows the structure of MASs-based microgrids. Current, energy storage, and others in microgrids have non-negative properties. It means that using PMASs is more suitable for modelling microgrid systems. At the same time, ETM can avoid infinite data transmission by the agent. In the model, *x*_*i*_(*t*), *u*_*i*_(*t*), *y*_*i*_(*t*), and *x*_0*k*_(*t*) denote the microgrid system state, resource scheduling control protocols, outputs, and leader component states, respectively. *A*, *B*, *C*, and *A*_0_ are the system matrices.

Consider the system (1) with observer:


$$\begin{array}{c} \begin{array}{c} A_{0}=(\begin{array}{cc} -1.89 & 1.92\\ 1.93 & -1.86 \end{array}),B_{0}=(\begin{array}{c} 0.41\\ 0.42 \end{array}),C_{0}=(\begin{array}{cc} 0.47 & 0.35 \end{array}),\\ A=(\begin{array}{cc} -0.95 & 0.97\\ 0.95 & -0.93 \end{array}),B=(\begin{array}{c} 0.42\\ 0.43 \end{array}),C=(\begin{array}{cc} 0.42 & 0.31 \end{array}). \end{array} \end{array}$$

**Fig 2:**
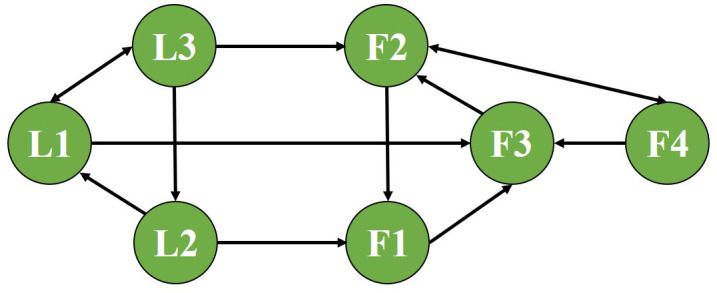
Communication topology.

Choose $\overset{―}{\alpha }=0.8$, $\overset{―}{\nu }=0.8$, $\overset{―}{\pi }=0.6$, $\overset{―}{\omega }=0.6$, $\varpi_{1}=0.3$, and $\varpi_{2}=0.5$. Fig 2 is communication topology.

By Theorem 2, one has


$$\begin{array}{c} \begin{array}{c} K_{01}=(\begin{array}{cc} -0.8936 & -0.6742 \end{array}),K_{02}=(\begin{array}{cc} -0.8936 & -0.6742 \end{array}),\\ K_{03}=(\begin{array}{cc} 0.8333 & 0.8531 \end{array}),K_{04}=(\begin{array}{cc} -1.0176 & -1.0337 \end{array}),\\ K_{05}=(\begin{array}{cc} -0.5010 & -0.3847 \end{array}),K_{06}=\begin{array}{c} 3.8456 \end{array},\\ G=(\begin{array}{cc} 0.7227 & 2.9953 \end{array}),K_{1}=(\begin{array}{cc} -0.2481 & -0.1886 \end{array}),\\ K_{2}=(\begin{array}{cc} -0.2595 & -0.2614 \end{array}),K_{3}=(\begin{array}{cc} 0.4050 & 0.4197 \end{array}),\\ K_{4}=(\begin{array}{cc} -0.3878 & -0.4593 \end{array}),K_{5}=(\begin{array}{cc} -0.2359 & -0.0442 \end{array}),\\ K_{6}=\begin{array}{c} 1.7033 \end{array},H=(\begin{array}{cc} 0.3972 & 1.5817 \end{array}),F_{0}=(\begin{array}{c} 0.4858\\ 0.4844 \end{array}),\\ L_{0}=(\begin{array}{c} -0.4045\\ -0.3951 \end{array}),F=(\begin{array}{c} 0.4285\\ 0.4267 \end{array}),L=(\begin{array}{c} -0.3135\\ -0.3022 \end{array}). \end{array} \end{array}$$

**Fig 3:**
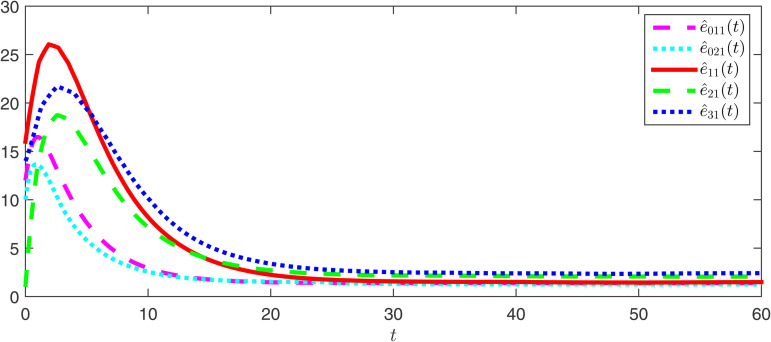
The simulations of the observer error ${\hat{e}}_{0k1}$ and ${\hat{e}}_{i1}$.

**Fig 4:**
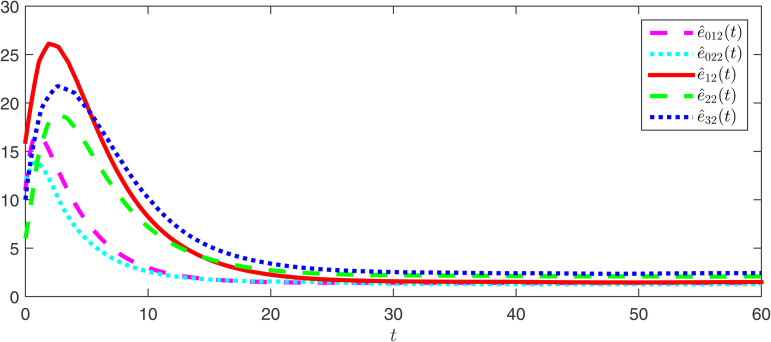
The simulations of the observer error ${\hat{e}}_{0k2}$ and ${\hat{e}}_{i2}$.

**Fig 5:**
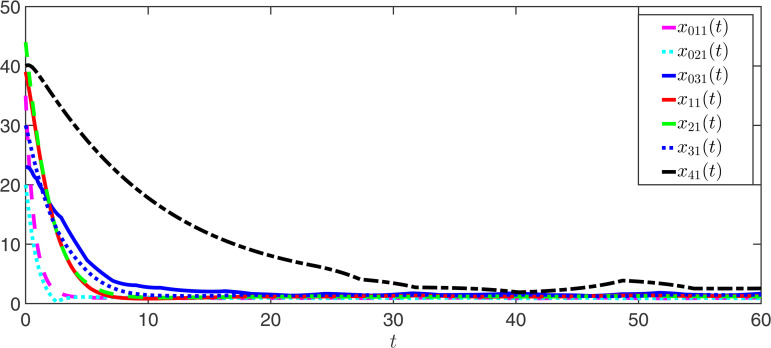
The simulations of the states *x*_0*k*1_ and *x*_*i*1_ under DAETC.

**Fig 6:**
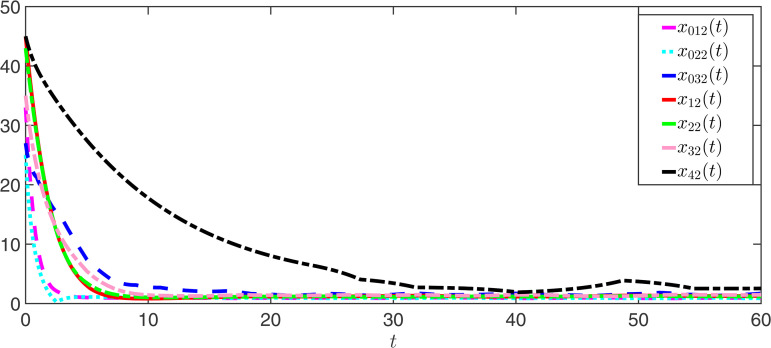
The simulations of the states *x*_0*k*2_ and *x*_*i*2_ under DAETC.

The observer error simulation curves of all agents are shown in Figs 3 and 4. It can be seen from the figures that the observer error of the state for all agents quickly stabilizes. This indicates that the positive observer proposed in Theorem 2 can effectively estimate the true state of the agents and is efficient. Figs 5 and 6 respectively draw the state trajectories of the leader and the follower under the multi-adaptive ETM. The figures clearly show that the state trajectories of the leader and followers eventually reach an acceptable region and remain stable. This also means that, under the designed observer and multi-adaptive event-triggered pinning controller, all agents achieve leader-follower consensus. This proves the validity of the proposed control scheme.

**Fig 7:**
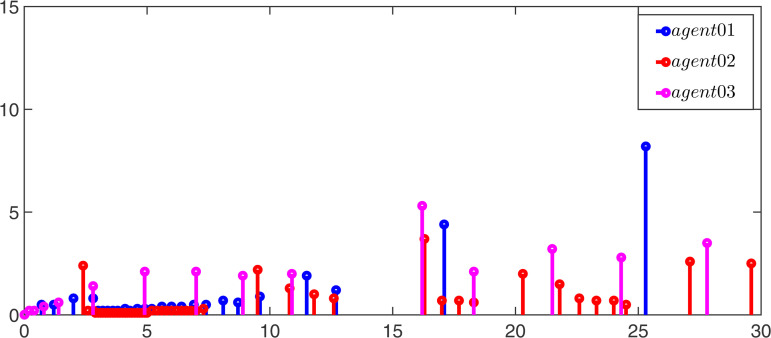
The event-triggered signal of the leaders’ controller.

**Fig 8:**
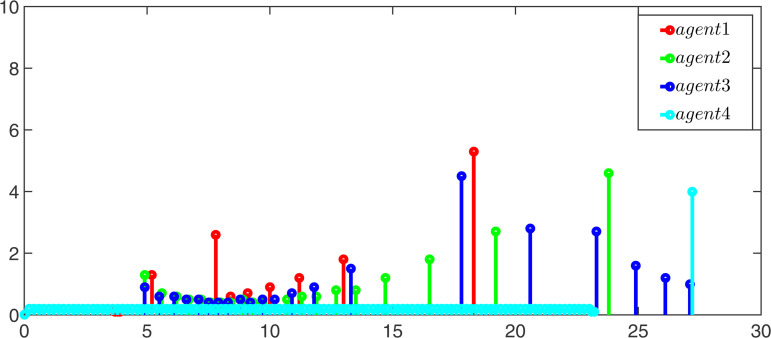
The event-triggered signal of the followers’ controller.

**Fig 9:**
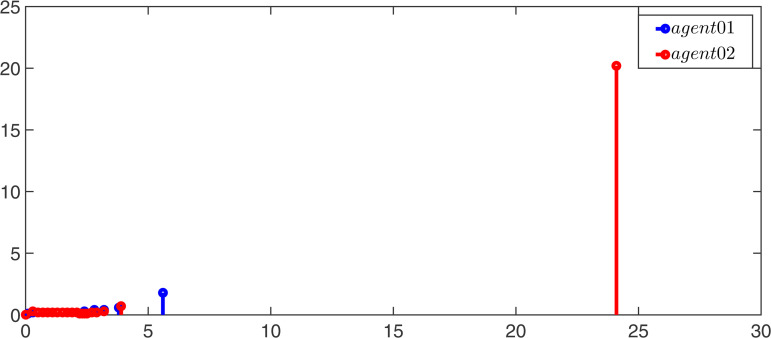
The event-triggered signal of the leaders’ observer.

**Fig 10:**
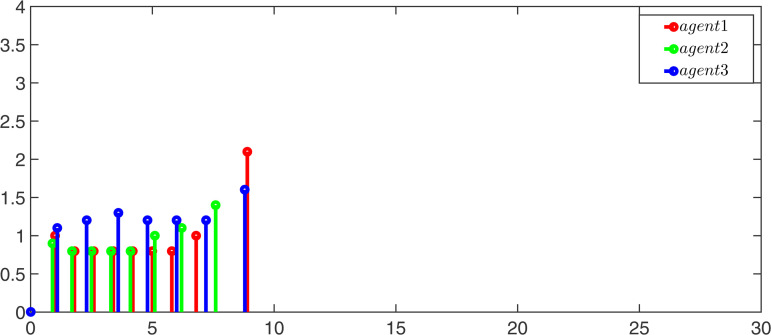
The event-triggered signal of the followers’ observer.

Moreover, the event-triggerred signals and intervals for observers and controllers of the leader and follower are displayed in Figs 7–10. According to the simulation results, it can be found that the multi-adaptive ETM proposed in this paper greatly reduces the update frequency of the controller and the observer, thereby saving communication resources.

To verify the robustness of the proposed consensus control protocol to faults and uncertainties, corresponding comparative simulations are performed. The steady-state errors of tracking errors *e*_0*k*_(*t*) and *e*_*i*_(*t*) are listed in Table 1.

**Table 1:** Performance comparisons.

Items			Steady-State Error	
			$\max\{e_{0k}(t)\}$	$\max\{e_{i}(t)\}$
Faults	Constant		**1.6755**	**3.5398**
	Attenuation		1.3849	3.4301
Uncertainty	Constant	Leader	**3.6848**	**3.3582**
		Follower	1.6703	9.9716
	Random	Leader	**2.8752**	**3.5173**
		Follower	1.6703	5.6064
Normal			1.6703	3.3540

(i) *Simulations under different faults:* Two types of faults are considered: constant faults and decaying faults, with values [0.8 0.8]^*T*^ and $[2e^{-0.2t}\;2e^{-0.2t}{]}^{T}$ respectively. Using the observer and controller in Theorem 2, the tracking errors between leaders as well as followers and the target are simulated. From the results recorded in Table 1, it can be observed that the steady-state error values of the tracking error under two different fault conditions are nearly identical to those under normal conditions. This indicates that the proposed consensus control protocol exhibits a certain degree of robustness against small-amplitude faults. It should be noted that this paper studies the practical consensus, therefore the steady-state value of the tracking error is not 0.
(ii) *Simulations under different uncertainty:* Assume that the system matrices *A*_0_ and *A* of leaders and followers have constant and random uncertainties, respectively. Consider the uncertain parameters of leaders and followers, and their corresponding values are chosen as follows: $c_{l}=0.08*ones(2)$, $c_{f}=0.03*ones(2)$, $r_{l}=0.08*rand(2,2)$, and $r_{f}=0.03*rand(2,2)$. Similarly, from the simulation results for uncertainty recorded in Table 1, it can be seen that when uncertainty exists in the leader, the steady-state value of the leader’s tracking error is significantly greater than under normal conditions, while the steady-state value of the follower’s tracking error remains essentially equivalent to that under normal conditions. When uncertainty exists among followers, the opposite occurs. These phenomena indicate that when uncertainty exists in the leader, the leader’s robustness is poor, while the follower’s control strategy shows some robustness. Conversely, when uncertainty exists in followers, the leader’s control strategy exhibits some robustness, while the follower’s control strategy shows poor robustness.

**Fig 11:**
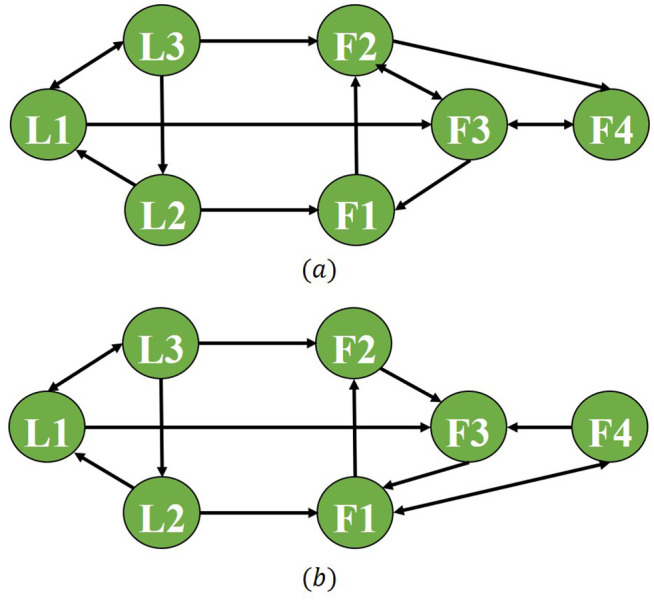
The switching network topology.

**Fig 12:**
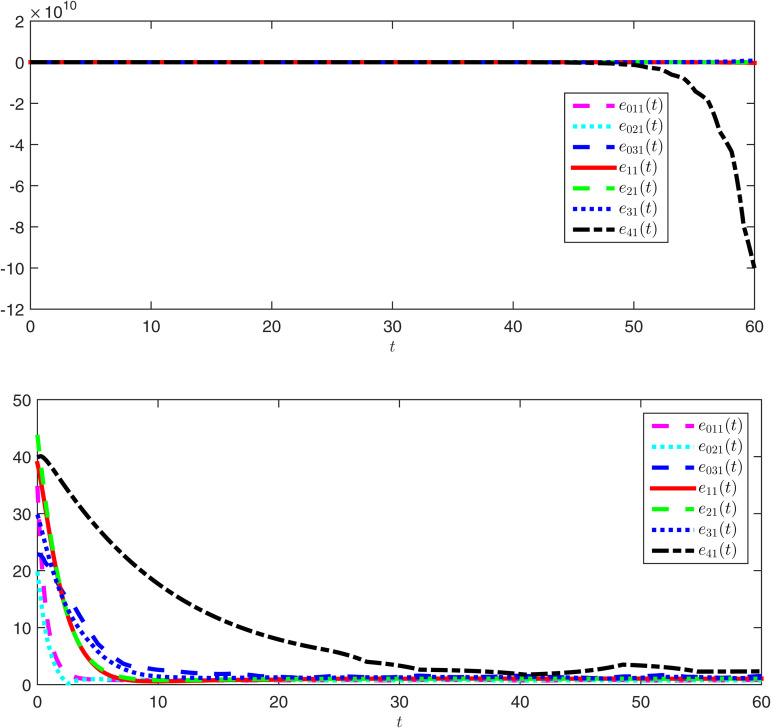
The responses of the tracking error under directed graph and switching topology.

To further validate the applicability of the proposed control scheme under complex network topologies, simulation results of the tracking error for agents under directed graphs and switching topology graphs are presented. Fig 11 shows the switched topology. The response curve of the tracking error between the follower agent and the target is plotted in Fig 12(a). Fig 12(b) presents the tracking error response curve under the directed graph (non-strongly connected). The simulation results reveal that the proposed event-triggered control scheme based on pinning strategy achieves practical consensus in the directed graph but fails to achieve consensus under switching topologies. This also indicates that future work should focus on further investigating the event-triggered pinning control problem under switching topologies.

Finally, we compared the update times of the proposed event-triggered pinning control strategy and the normal control strategy. As can be seen from Table 2, the update times of the proposed event-triggered traction controller is much lower than that of the normal controller, which also proves the effectiveness and superiority of the proposed multi-adaptive event-triggered pinning control scheme.

**Table 2:** Comparison of update times between Normal controller and Pinning controller.

Items	Normal controller for followers				Pinning controller (30) for followers			
	F1	F2	F3	F4	F1	F2	F3	F4
Update times	600	600	600	600	27	35	45	122

## 6 Conclusion

In this paper, the multi-adaptive event-triggered consensus of PMASs has been investigated based on pinning control. First, adaptive event-triggered pinning control protocols were designed for the leader and follower systems, and ETMs were constructed for leaders and followers, respectively. Under such protocols, the considered systems achieved practical consensus. Then, an adaptive event-triggered observer, intended only for the pinned agent, was proposed to estimate its state. Subsequently, an observer-based adaptive event-triggered control protocol was constructed, resulting in a closed-loop system composed of observation and tracking errors. Finally, conditions ensuring the positivity and consensus of the closed-loop system were established using LP and CLF. Compared to the proposed conclusions, the proposed scheme not only significantly reduces the control cost and resource consumption of the system but also improves the practicality and stability of the system.

In future work, the pinning control can be applied to other positive systems, such as positive complex networks, positive fuzzy MASs, etc. Moreover, factors such as the choice of multiple leaders, communication delay, uncertain parameters, quantization effects, and measurement noise are also topics worthy of in-depth study in PMASs.
